# Metabolic priming of GD2 *TRAC*-CAR T cells during manufacturing promotes memory phenotypes while enhancing persistence

**DOI:** 10.1016/j.omtm.2024.101249

**Published:** 2024-04-10

**Authors:** Dan Cappabianca, Dan Pham, Matthew H. Forsberg, Madison Bugel, Anna Tommasi, Anthony Lauer, Jolanta Vidugiriene, Brookelyn Hrdlicka, Alexandria McHale, Quaovi H. Sodji, Melissa C. Skala, Christian M. Capitini, Krishanu Saha

**Affiliations:** 1 Wisconsin Institute for Discovery, University of Wisconsin-Madison, Madison, WI 53715, USA; 2 Department of Biomedical Engineering, University of Wisconsin-Madison, Madison, WI 53715, USA; 3 Morgridge Institute for Research, University of Wisconsin-Madison, Madison, WI 53715, USA; 4 Department of Pediatrics, University of Wisconsin School of Medicine and Public Health, Madison, WI 53705, USA; 5 Promega Corporation, Fitchburg, WI 53711, USA; 6 University of Wisconsin Carbone Cancer Center, University of Wisconsin-Madison, Madison, WI 53705, USA; 7 Department of Human Oncology, University of Wisconsin School of Medicine and Public Health, Madison, WI 53705, USA

**Keywords:** gene editing, CAR T cells, cancer, metabolism, stem cell memory, CRISPR, GMP-compatible, pre-clinical, scale-up, biomanufacturing

## Abstract

Manufacturing chimeric antigen receptor (CAR) T cell therapies is complex, with limited understanding of how medium composition impacts T cell phenotypes. CRISPR-Cas9 ribonucleoproteins can precisely insert a CAR sequence while disrupting the endogenous T cell receptor alpha constant (*TRAC*) gene resulting in *TRAC*-CAR T cells with an enriched stem cell memory T cell population, a process that could be further optimized through modifications to the medium composition. In this study we generated anti-GD2 *TRAC*-CAR T cells using "metabolic priming" (MP), where the cells were activated in glucose/glutamine-low medium and then expanded in glucose/glutamine-high medium. T cell products were evaluated using spectral flow cytometry, metabolic assays, cytokine production, cytotoxicity assays *in vitro*, and potency against human GD2+ xenograft neuroblastoma models *in vivo*. Compared with standard *TRAC*-CAR T cells, MP *TRAC*-CAR T cells showed less glycolysis, higher CCR7/CD62L expression, more bound NAD(P)H activity, and reduced IFN-γ, IL-2, IP-10, IL-1β, IL-17, and TGF-β production at the end of manufacturing *ex vivo*, with increased central memory CAR T cells and better persistence observed *in vivo*. MP with medium during CAR T cell biomanufacturing can minimize glycolysis and enrich memory phenotypes *ex vivo*, which could lead to better responses against solid tumors *in vivo*.

## Graphical abstract

**Figure:**
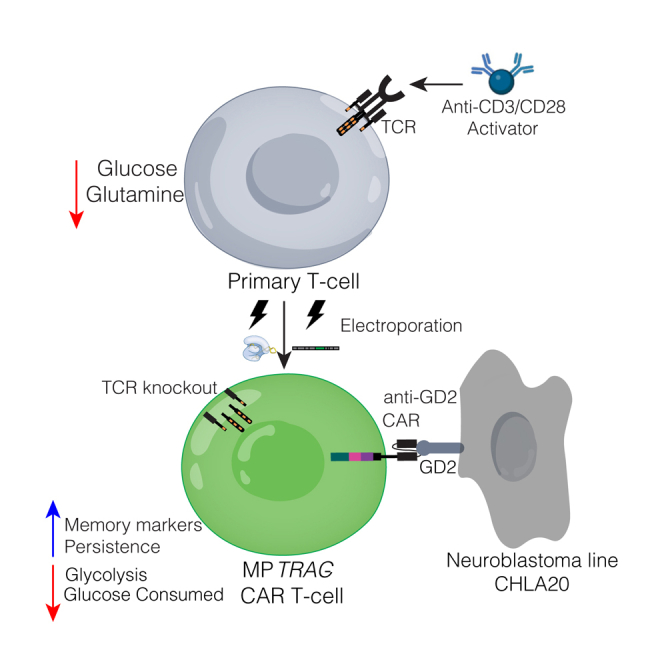

## Teaser

Cappabianca and colleagues manufactured virus-free CAR T cells at scale with CRISPR-Cas9 and “metabolically primed” them by attenuating activation in low-glucose/glutamine medium with expansion in high-glucose/glutamine medium. Priming made CAR T cells with increased stem cell memory properties, including enriched central memory phenotypes *in vivo* while lysing solid tumors.

## Introduction

Chimeric antigen receptor (CAR) T cells have emerged as an exciting alternative to traditional cancer treatments for hematologic malignancies with six FDA-approved therapies for multiple myeloma, non-Hodgkin B cell lymphomas, and B cell acute lymphoblastic leukemia available to date.^1^ In contrast, CAR T therapies for solid tumors have had limited clinical responses due to a lack of persistence, limited homing to the tumor, and exhausted T cell phenotypes.^2^^,^^3^ Clinically, neuroblastoma initially demonstrated the potential efficacy of CAR T cells in treating solid tumors. However, a subsequent analysis revealed a modest 15% response rate with durable responses only achieved in patients whose GD2 CAR T cells persisted longer than 6 weeks after infusion and formed central memory T cells.^4^^,^^5^ Recently, promising results for advanced neuroblastoma with a third-generation anti-GD2 CAR T cell were reported, with a 63% partial or complete response rate; however, event-free survival remained low at 26%.^6^ The critical quality attributes that contribute to CAR T cell persistence and central memory formation in solid tumors remain largely unknown.

Manufacturing CAR T cells to reach therapeutic cell quantities (∼0.1–10 billions cells per patient) involves inducing cell proliferation *ex vivo* through activation with crosslinked antibodies for CD3 and CD28, and cytokine-enriched medium.^7^^,^^8^^,^^9^ *Ex vivo* culture of CAR T cells has been previously shown to accelerate differentiation.^10^^,^^11^^,^^12^ Limiting differentiation of these cells into terminal effector or exhausted phenotypes pre-infusion is a goal for the field, since increased stem cell memory fractions in the pre-infusion product have correlated with better cytotoxicity post-infusion, transition to a central memory state, and better persistence *in vivo*.^13^^,^^14^^,^^15^^,^^16^^,^^17^^,^^18^^,^^19^^,^^20^ Enhanced stem cell or central memory phenotypes in CAR T products could be achieved by manipulating media, cytokines, or T cell metabolism during manufacturing. Tailored media and IL-7/IL-15-based expansion have been found to preserve a stem cell memory profile.^21^ Metabolic interventions that maintain oxidative phosphorylation (OXPHOS) and suppress glycolysis through metabolic engineering,^22^^,^^23^^,^^24^ small-molecule inhibitors, or glucose/glutamine deprivation^25^^,^^26^^,^^27^^,^^28^^,^^29^ have been shown to enhance the cell persistence, potency, and memory formation of CAR T products. These strategies have yet to be explored with CAR T cell products that have been genome-edited using electroporation (EP) in a virus-free manner.

T cells have classically been transduced with γ-retroviral or lentiviral vectors yielding stable but uncontrolled genomic integration of the CAR transgene with a constitutively active promoter,^30^^,^^31^ which has been implicated in excessive T cell differentiation.^32^ CRISPR-Cas9 is an alternative approach that makes a double-stranded DNA (dsDNA) break at a precise genomic locus where the DNA repair pathways can stably integrate the desired CAR transgene.^33^^,^^34^^,^^35^ This genome editing strategy has been used to insert a CAR transgene upstream of the endogenous T cell receptor alpha constant (*TRAC*) gene using homology-directed repair (HDR).^36^^,^^37^^,^^38^ For current *TRAC* integration strategies, the CAR T cell products lack an intact T cell receptor (TCR) because of the precise genetic knockout of the TCR α chain. Relative to conventional viral CAR T products, these *TRAC*-CAR products have more controlled transgene copy numbers in the genome (1 or 2) with CAR transcription driven by the *TRAC* promoter, limited off-target effects, and higher fractions of stem cell memory phenotypes.^39^^,^^40^^,^^41^ A benchtop-scale CAR T cell process for inserting an anti-GD2 construct into the *TRAC* locus using EP of CRISPR-Cas9 ribonucleoproteins (RNPs) with PCR-based donor templates showed promising results in a GD2^+^ human neuroblastoma xenograft model.^41^ Many studies have optimized culture conditions for CAR T cells manufactured using viral vectors,^17^^,^^21^^,^^25^ but it is unknown whether these culture changes will affect *TRAC*-CAR T cells generated by EP in a similar manner.

In this study, we evaluated various culture conditions to develop a flexible process using GMP-compatible reagents for producing *TRAC*-CAR T cells at scales suitable for clinical use. We focused on metabolic priming (MP), where T cells are activated in TexMACS medium and then switched post-EP to Immunocult XF. TexMACS medium is associated with attenuated cellular activation, possibly due to its lower glucose/glutamine content relative to Immunocult XF.^17^^,^^42^ The result is that MP *TRAC*-CAR T cells are less reliant on glycolysis, exhibit more stem cell memory phenotypes, and consume less glucose than their non-primed counterparts. Furthermore, these MP *TRAC*-CAR T cells can adopt a central memory phenotype following exposure to solid tumors in neuroblastoma xenograft models. This biomanufacturing approach thus holds promise for generating cells with enhanced potency and longevity *in vivo*.

## Results

### *TRAC*-CAR T cell manufacturing at scale

**Figure 1:**
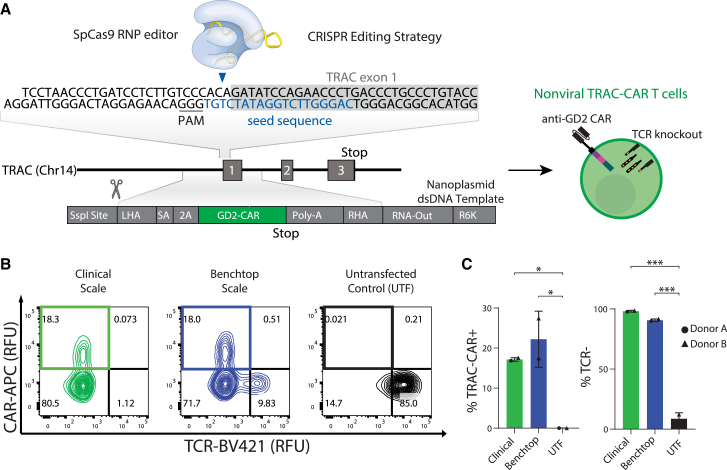
Manufacturing *TRAC*-CAR T cells at clinically relevant scales (A) Schematic of non-viral CRISPR-Cas9 knockin strategy with a Nanoplasmid HDR template encoding the CAR. The CAR-containing Nanoplasmid template encodes for an anti-GD2 CAR under the control of the endogenous *TRAC* promoter. The CAR-containing Nanoplasmid template has an SSPI restriction site for linearization. (B) Representative flow cytometry plots of the relative GD2-CAR versus TCR expression of *TRAC*-CAR T cells electroporated with SSPI-linearized nanoplasmid on the ThermoFisher Xenon (clinical scale) or Lonza 4D Nucleofector (benchtop scale) system and untransfected T cells (donor A shown). (C) Bar graphs comparing GD2 knockin rates and TCR^−^ percentages of *TRAC*-CAR T cells electroporated on the clinical or benchtop systems and untransfected T cells. Two donors, N_Clinical_ = N_Benchtop_ = N_Untransfected_ = 2. SA, splice acceptor; 2A, cleavage peptide; LHA, left homology arm; RHA, right homology arm; Poly-A, rabbit β-globin polyA terminator; CAR, chimeric antigen receptor; *TRAC*, T cell receptor alpha constant gene; RNP, ribonucleoprotein; PAM, protospacer adjacent motif; TCR, T cell receptor; RFU, relative fluorescence units. Error bars represent mean and standard deviation. Statistical significance was determined with a one-way ANOVA using Dunnett’s T3 test for multiple comparisons (C); ∗p < 0.05, ∗∗∗p < 0.001.

CAR T cells ideally should proliferate *ex vivo* to achieve clinically relevant yields, while maintaining a naive, stem cell memory/central memory state to achieve persistence *in vivo.*^10^^,^^14^^,^^43^ We first explored whether a dsDNA Nanoplasmid template could provide a facile method to scale-up production of the donor template for HDR to generate *TRAC*-CAR T cells (Figure 1A). Nanoplasmid vectors have a small backbone (429 bp) and no antibiotic selection genes. They also contain a specialized bacterial R6K replication origin in place of the traditional pUC origin, making the Nanoplasmid vectors replication-incompatible outside of a set of engineered strains. Minimal-backbone Nanoplasmid transposon vectors and Nanoplasmid HDR donor template vectors for CRISPR/Cas9 mediated gene editing demonstrated superior cell viability and gene integration frequencies during non-viral CAR-T cell manufacturing over traditional pUC plasmids and linearized templates (Clinical Trial NCT03288493).^55^^,^^64^^,^^65^^,^^66^ We electroporated this template along with a CRISPR-Cas9 RNP specific for the *TRAC* locus and cultured the cells for 7 days. Robust gene editing at clinical or benchtop scale was observed by flow cytometry, as greater than 90% of T cells lacked expression of the endogenous TCR given the CRISPR-mediated knockout of the *TRAC* gene (Figure 1B) when compared with untransfected T cells (clinical: 98.2% [0.7] or benchtop: 90.7% [1.0] versus untransfected: 8.6% [5.1], p < 0.001, respectively). *TRAC*-CAR T cells were produced equally well at both scales with over 17% CAR positivity using the Lonza or Xenon EP systems (clinical: 17.1% [0.4] or benchtop: 22.2% [7] versus untransfected: 0.02% [0.01], p = 0.024 and p = 0.049, respectively) (Figure 1C).

### MP to enrich for stem cell memory

**Figure 2:**
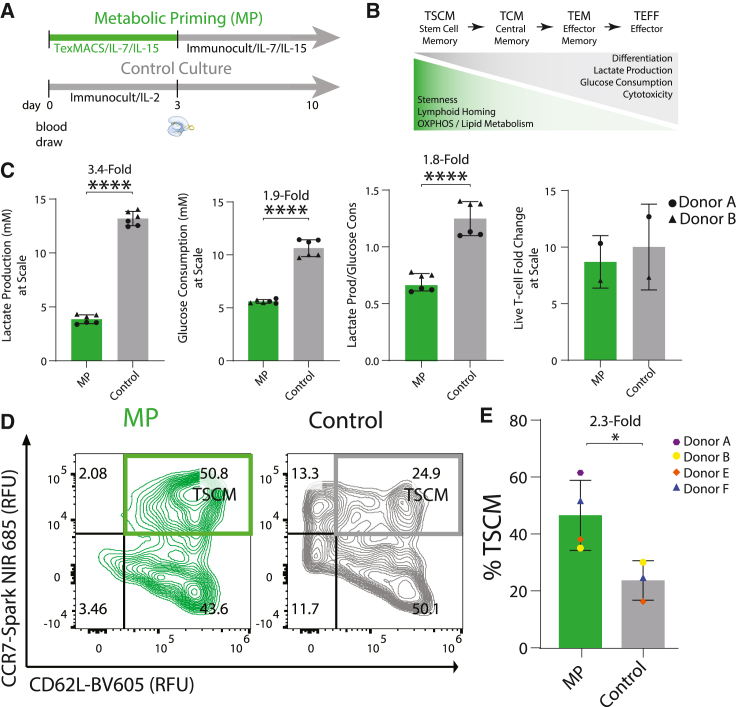
MP *TRAC*-CAR T cells produce stem cell memory phenotypes (A) Manufacturing timeline for *TRAC*-CAR T cells: MP *TRAC-*CAR T cells were cultured in TexMACS medium for 3 days during activation and then Immunocult XF medium supplemented with IL-7/IL-15 for 7 days during expansion. Control *TRAC*-CAR T cells were cultured in only Immunocult XF medium supplemented with IL-2 during activation and expansion. (B) Schematic of the definition of CAR T cell phenotypes: T_SCM_ (stem cell memory), T_CM_ (central memory), T_EM_ (effector memory), T_EFF_ (effector) as defined by their inherent properties. (C) Bar graphs of the lactate production, glucose consumption, lactate production over glucose consumption, and live T cell fold-change of MP or Control *TRAC*-CAR T cells during expansion in G-Rex6M plates. Two donors, (lactate/glucose) N_MP_ = N_Control_ = 6; (proliferation) N_MP_ = N_Control_ = 2. (D) Representative contour plots of CD62L/CCR7 co-expression for CAR^+^/TCR^−^ MP and Control *TRAC*-CAR T cells for the same conditions. CD62L/CCR7 double-positive cells represent a stem cell memory population (T_Scm_) (donor F shown). (E) Bar graph depicting the CD62L/CCR7 double-positive population in CAR^+^ T cells for all conditions. Four donors, N_MP_ = 4, N_Control_ = 3. Error bars represent mean and standard deviation. Statistical significance was determined with a paired t test (C), and Welch’s t test (E); ∗p < 0.05, ∗∗∗∗p < 0.0001.

Generally, CAR T cell production uses a singular medium source and cytokine cocktail throughout manufacturing to expand T cells into clinically relevant numbers *ex vivo*. Activation and expansion of *TRAC*-CAR T cells has previously been performed using Immunocult XF medium supplemented with IL-2,^41^ generating stem cell memory T cells with high expression of CD45RA, CD62L, and CCR7 and CAR knockin rates between 15% and 34%.^13^^,^^44^ We sought to optimize culture conditions that could further increase the proportion of stem cell memory *TRAC*-CAR T cells by manufacturing cells in two media conditions (Figure 2A) using reagents produced via good manufacturing practice (GMP) at a clinically relevant scale: (1) standard Immunocult XF Medium^17^ with IL-2 for 10 days (Control cells) or (2) a transient MP phase, where cells were activated in TexMACS with IL-7/IL-15^21^^,^^45^ before EP and then expanded in Immunocult XF with IL-7/IL-15 for 7 days post-EP (MP cells).

Memory T cells primarily use OXPHOS and fatty acid oxidation for long-term survival, whereas effector T cells rely on glycolysis for rapid proliferation and immediate immune response.^22^^,^^23^^,^^24^ In cell cultures, a lower glucose consumption and lactate secretion may indicate a higher proportion of stem cell memory cells (Figure 2B).^22^^,^^23^^,^^24^ We measured the lactate and glucose concentrations of MP and Control *TRAC-*CAR T cells immediately after activation on day 3 and on day 10 of manufacturing to assess their stem cell memory properties. Post-activation MP *TRAC-*CAR T cells produced less lactate (MP: 4.2 mM [2.2] versus Control: 9.2 mM [0.3], p = 0.002) and consumed less glucose (MP: 3.6 mM [2.4] versus Control: 13.2 mM [0.5], p < 0.001) despite equal fold expansion during activation (MP: 1.1 [0.3] versus Control: 1.2 [0.02]) (Figure S1). During expansion MP *TRAC*-CAR T cells had produced 1.8-fold less lactate per glucose molecule (MP: 0.7 [0.1] versus Control: 1.3 [0.1], p < 0.001), 3.4-fold less overall lactate (MP: 3.9 mM [0.4] versus Control: 13.2 mM [0.6], p < 0.001), and consumed 1.9-fold less glucose (MP: 5.6 mM [0.2] versus Control: 10.6 mM [0.7], p < 0.001) than Control *TRAC-*CAR T cells during expansion in G-Rex6M plates despite having similar yields of cells by day 10 (MP: 8.7 [1.6] versus Control: 10 [2.7]) (Figure 2C), indicating that MP does not affect cell yield in this process.

We also demonstrated the importance of transitioning from TexMACs to Immunocult XF with IL-7/IL-15 versus continued culture in TexMACs as the former had the least lactate production and glucose consumption among *TRAC-*CAR T cells manufactured with MP, TexMACs only, or Immunocult XF only supplemented with IL-7/IL-15 or IL-2 (Figure S2). MP *TRAC-*CAR T cells also expanded 1.8 times more than those grown in TexMACs only (Figure S3).

To specifically analyze the phenotypes of the CAR-positive cells in the cell product on day 10, we stained for surface markers of T cell differentiation and memory phenotypes using a spectral flow cytometry panel and analyzed live CAR^+^/TCR^−^ cells (Figure S4). We classified T cell phenotypes by classic definitions: T_SCM_ (stem cell memory), T_CM_ (central memory), T_EM_ (effector memory), and T_EFF_ (effector). A stem cell memory state is often defined by the expression of surface markers such as CD45RA, CD62L, and CCR7^13^^,^^46^ an oxidative versus a glycolytic metabolism, commonly seen in activated effector T cells,^18^^,^^47^ and lower glucose consumption^29^^,^^48^^,^^49^ (Figure 2B). We defined T_SCM_ *TRAC-*CAR T cells as CCR7^+^/CD62L^+^, which indicates increased capacity for lymphoid homing.^50^^,^^51^ *TRAC*-CAR T cells grown in Immunocult XF are often CD45RA/CD45RO double-positive, likely reflecting a transitional state that cannot be assigned to canonical memory subsets.^39^^,^^41^ This transitional phenotype is present in both MP and Control *TRAC-*CAR T cells (Figure S5). We found that MP *TRAC-*CAR T cells produced 2.3-fold more CD62L^+^/CCR7^+^ CAR^+^ T cells than Control *TRAC-*CAR T cells (MP: 46.9% [12.3] versus Control: 23.9% [7.0], p = 0.028) (Figures 2D and 2E). We also observed no significant impacts of priming on CD8 or CD4 expression (Figure S5). *TRAC-*CAR T cells cultured in TexMACs and MP *TRAC*-CAR T cells had similar CD62L/CCR7 expression that did not appear impacted by cytokines (Figure S2).

### Priming lowers glycolysis and effector phenotypes

**Figure 3:**
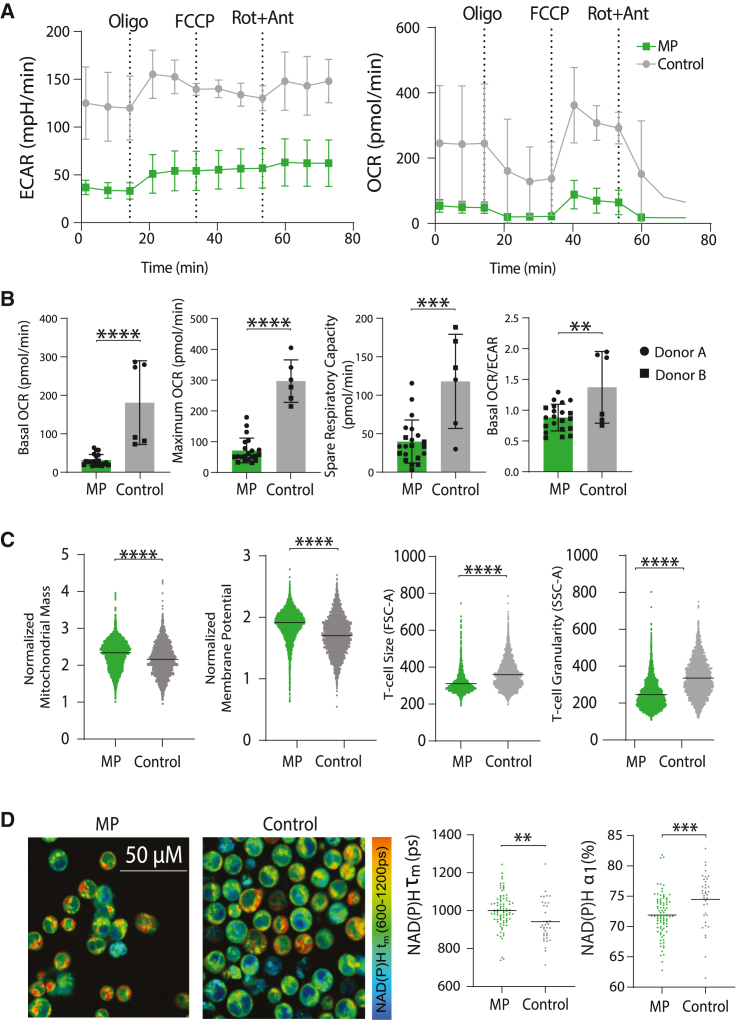
MP *TRAC-*CAR T cells are less glycolytic and have increased mitochondrial mass (A) ECAR and OCR for MP and Control *TRAC-*CAR T cells measured over time by Seahorse (oligomycin [2.5 μM], FCCP [1 μM], rotenone/antimycin A [0.5 μM]). (B) Basal/maximum respiration rates, spare respiratory capacity, and basal OCR/ECAR for MP and Control *TRAC*-CAR T cells measured by Seahorse. Two donors, N_MP_ = 21, N_Control_ = 6. (C) Dot plots for normalized mitochondrial mass (MitoTracker Green intensity divided by FSC-A), normalized mitochondrial membrane potential (TMRE dye intensity divided by FSC-A), cell size (FSC-A), and granularity (SSC-A) for CAR^+^ MP and Control *TRAC*-CAR T cells. Two donors, N_MP_ = 4264, N_Control_ = 2439. (D) Images and bar graphs of NAD(P)H mean lifetime (NAD(P)H τ_m_) and free NAD(P)H fraction (NAD(P)H α_1_) of CAR^+^ MP and Control *TRAC*-CAR T cells as measured by fluorescence lifetime imaging. Two donors, N_MP_ = 84, N_Control_ = 37. OCR, oxygen consumption rate; ECAR, extracellular acidification rate; SSC-A, side scatter area; FSC-A, forward scatter area; TMRE, tetramethylrhodamine ethyl ester perchlorate. Error bars represent mean and standard deviation. Statistical significance was determined with unpaired t tests; ∗∗p < 0.01, ∗∗∗p < 0.001, ∗∗∗∗p < 0.0001.

To further characterize metabolism, we harvested cells on day 10 of manufacturing for use in a Seahorse assay to analyze oxygen consumption and extracellular acidification rates and for mitochondrial staining by flow cytometry. MP *TRAC-*CAR T cells had lower rates of ECAR (extracellular acidification) and OCR (oxygen consumption) than Control *TRAC-*CAR T cells, indicating lower rates of both glycolysis and overall oxygen consumption (Figure 3A). Lower basal respiration (MP: 32 pmol/min [14] versus Control: 180 pmol/min [99], p < 0.001), maximal respiration (MP: 72 pmol/min [39] versus Control: 298 pmol/min [63], p < 0.001), spare respiratory capacity (MP: 40 pmol/min [27] versus Control: 117 pmol/min [56], p < 0.001), and basal OCR/ECAR (MP: 0.88 [0.21] versus Control: 1.37 [0.53], p = 0.0033) were seen in MP compared with Control *TRAC-*CAR T cells (Figure 3B). In addition, MP *TRAC-*CAR T cells had 6% higher normalized mitochondrial mass (MP: 2.3 [0.4] versus Control: 2.2 [0.4], p < 0.001, d = 0.33) and 11% higher membrane potential (MP: 1.9 [0.2] versus Control: 1.7 [0.3], p < 0.001, d = 0.64) than Control *TRAC-*CAR T cells, further supporting an oxidative phenotype (Figure 3C). A 27% reduction in granularity (MP: 246 [76] versus Control: 336 [98], p < 0.001, d = 1.02) and 14% lower cell size (MP: 310 [52] versus Control: 360 [78], p < 0.001, d = 0.74) indicates that MP *TRAC-*CAR T cells may be in a less cytotoxic state^52^ after manufacturing than Control *TRAC-*CAR T cells (Figure 3C).

To resolve metabolism at single-cell resolution and assess heterogeneity, we also performed fluorescence lifetime imaging^62^^,^^63^ of *TRAC-*CAR T cells after manufacturing. We specifically measured autofluorescence lifetimes of NAD(P)H and FAD and report the NAD(P)H mean fluorescence lifetime (NAD(P)H τ_m_) and free NAD(P)H fraction (NAD(P)H α_1_) of CAR^+^ *TRAC-*CAR T cells, which reflect NAD(P)H binding activity. MP *TRAC-*CAR T cells had higher NAD(P)H τ_m_ (MP: 1,000 ps [93] versus Control: 942 ps [110], p = 0.003) and lower NAD(P)H α_1_ (MP: 72% [4] versus Control: 75% [4], p < 0.001) than Control-*TRAC-*CAR T cells indicating a higher proportion of bound NAD(P)H (Figure 3D) in MP *TRAC*-CAR T cells. These label-free, single-cell analyses confirm the presence of more primed cells with increased NAD(P)H binding activity within the MP *TRAC*-CAR T cell products and are consistent with lifetime imaging of similar *TRAC-*CAR products.^42^

### High potency of MP *TRAC*-CAR T cell products

**Figure 4:**
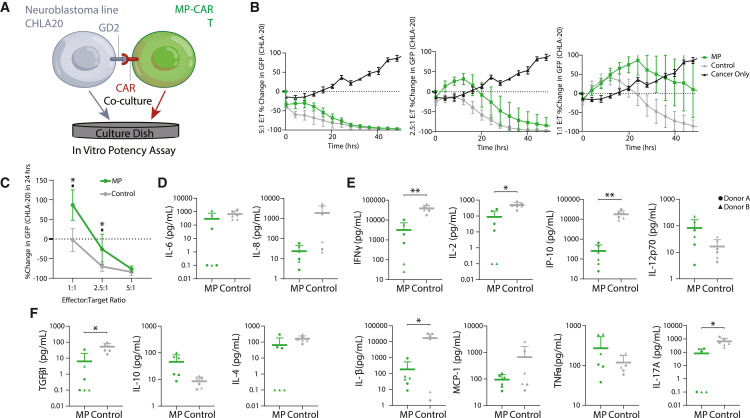
MP *TRAC-*CAR T cells have reduced cytokine production but are equipotent *in vitro* against GD2^+^ neuroblastoma cells (A) GD2^+^ neuroblastoma CHLA-20 cells were plated in 24- or 96-well plates 24 h before *TRAC*-CAR T cell addition. The supernatant was analyzed for cytokine secretion after 24 h, and potency was measured continuously for up to 48 h. (B) Percentage change in GFP fluorescence from GD2^+^ CHLA-20 GFP^+^ neuroblastoma cells versus time in cancer/MP or Control *TRAC*-CAR T cell co-cultures for E:T ratios of 5:1, 2.5:1, or 1:1. (C) The percentage change in GFP signal at 24 h versus E:T ratio is shown for MP or Control *TRAC*-CAR T cells. Co-culture supernatant was analyzed using the LegendPlex Human Essential Immune Response (BioLegend) assay. (D) Pleiotropic, (E) Pro-inflammatory and (F) Anti-inflammatory secreted cytokine concentrations normalized to live T cell count after co-culture depicted on histograms on log scale (pg/mL per 1e–6 T cells). GFP, green fluorescent protein. Two donors, N_MP_ = 6, N_Control_ = 6. Error bars represent mean and standard deviation. Statistical significance was determined with paired t tests; ∗p < 0.05, ∗∗p < 0.01.

To assess the potency of the MP *TRAC-*CAR T cells, we measured cytotoxicity and cytokine production after co-culture with the GD2^+^ neuroblastoma cell line, CHLA20. These target cells were seeded at various effector:target (E:T) ratios into 24- or 96-well plates and grown for 24 h, after which *TRAC-*CAR T cell products were added. The supernatant was taken at 24 h, and the co-culture was imaged continuously for 48 h on the IncuCyte platform (Figure 4A). MP and Control *TRAC-*CAR T cells achieved similar extents of cytotoxicity for 5:1 and 2.5:1 E:T ratios (Figure 4B). After 24 h of co-culture, MP *TRAC-*CAR T cells lysed fewer cancer cells than Control *TRAC*-CAR T cells at 2.5:1 (MP: −26% [38.1] versus −70.2% [12.1], p = 0.041) and 1:1 (MP: 86.5% [38.8] versus Control: −2.5% [29.3], p = 0.011) ratios (Figure 4C), but had similar levels of cytotoxicity at the 5:1 ratio. In terms of cytokine production, soluble IFN-γ (MP: 3,151 pg/mL [4,119] versus Control: 38,453 pg/mL [16,037], p = 0.0045), IL-2 (MP: 88 pg/mL [121] versus Control: 496 pg/mL [229], p = 0.0195), IP-10 (MP: 254 pg/mL [257] versus Control: 17,853 pg/mL [8,494], p = 0.004), IL-1β (MP: 185 pg/mL [358] versus Control: 16,882 pg/mL [14,578], p = 0.0372), IL-17 (MP: 81 pg/mL [93] versus Control: 664 pg/mL [460], p = 0.0323), and TGF-β (MP: 6.4 pg/mL [13.7] versus Control: 51.9 pg/mL [32.2], p = 0.0338) levels produced by MP *TRAC-*CAR T cells in the media were significantly lower than Control *TRAC*-CAR T cells while all other measured cytokines had no differences (Figures 4D–4F). Compared with Control *TRAC*-CAR T cells, MP *TRAC-*CAR T cells have comparable but slower cytotoxic activity with overall lower cytokine production immediately following antigen stimulation *in vitro.* These characteristics have been observed in less differentiated cells, notably T_SCM_ cells.^13^^,^^49^

To assess the impact of these quality attributes on potency *in vivo*, *TRAC-*CAR T cells of both groups were infused into a xenograft NSG mouse model of neuroblastoma. There was similar tumor regression across the two groups (Figure S6), indicating comparable *in vivo* potency of cells from both culture conditions. Despite lower levels of initial cytotoxicity and cytokine release *in vitro*, MP *TRAC-*CAR T cells demonstrate high potency against solid tumors *in vivo.* Because they were less active in culture, it was unclear if these attributes would make them less prone to exhaustion and exhibit better persistence and central memory formation *in vivo.*

### Enhanced central memory and persistence *in vivo*

**Figure 5:**
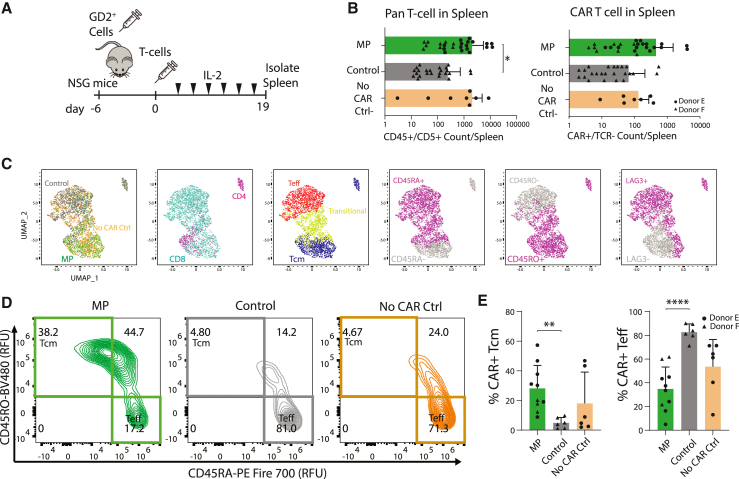
MP *TRAC-*CAR T cells display a central memory phenotype and better persistence *in vivo* after treatment of GD2+ neuroblastoma (A) NSG mice were injected with CHLA-20 cells 1 week before *TRAC-*CAR T injection, upon which mice were treated with 3 million CAR^+^ cells by tail vein injection. IVIS imaging was performed every 3–4 days with IL-2 supplementation via the tail vein. Mouse spleens were isolated on day 19 post-injection and stained to immunophenotype human T lymphocyte via flow cytometry. (B) Bar graphs depicting the number of CD5/CD45^+^ events from isolated mouse spleens (left) and CAR^+^ or mCherry^+^/TCR^−^ lymphocytes within that population for mice treated with MP *TRAC-*CAR T, Control *TRAC*-CAR T, or No CAR Control (mCherry) cells (right) on log scale. Two donors, N_MP_ = 26, N_Control_ = 24, N_NoCARCtrl_ = 8. (C) Marker expression UMAPs of MP, Control *TRAC*-CAR T cells, or No CAR Control T cells. These maps were generated using flow cytometry to track CD45RA, CD45RO, CD62L, CCR7, LAG3, and TIGIT levels. Dot plots separate cells by condition, CD4/CD8 levels, memory or effector status (CD45RA^−^/CD45RO^+^ [central memory (T_cm_)], CD45RA^+^/CD45RO^−^ (effector (T_eff_)], or CD45RA^+^/CD45RO^+^ [transitional T cells]), CD45RO, CD45RA, or LAG3 levels. (D) Representative contour plots of the levels of CD45RA versus CD45RO separate MP *TRAC-*CAR T (donor E), Control *TRAC*-CAR T (donor F), and No CAR Control cells (donor E) by central memory or effector status. (E) Bar graphs show the relative expression of cell populations for mice with greater than 20 transgene^+^/TCR^−^ events in the spleen: T_cm_ or T_eff_. Two donors, N_MP_ = 10, N_Control_ = 6, N_NoCARCtrl_ = 6. Error bars represent mean and standard deviation. Statistical significance was determined with Brown-Forsythe and Welch ANOVA tests using Dunnett’s T3 test for multiple comparisons; ∗p < 0.05, ∗∗p < 0.01, ∗∗∗∗p < 0.0001.

To assess memory phenotypes and persistence of MP *TRAC-*CAR T cells *in vivo*, we injected T cell products into a xenograft NSG mouse model for neuroblastoma (Figure 5A). Groups included MP *TRAC*-CAR or Control *TRAC-*CAR cells and No CAR Control cells, which are *TRAC-*T cells expressing an mCherry reporter gene instead of CAR and cultured in the standard Control conditions. On day 19 post-injection, we isolated splenocytes, and stained samples for spectral flow cytometry analysis of memory, activation, and exhaustion markers (Figure S7). We found significantly higher amounts of human CD5/CD45^+^ (MP: 2,025 cells [3,433], Control: 244 cells [487], No CAR Ctrl: 2,055 [2,841], p [MP versus Control] = 0.0423) amounts of lymphocytes in spleens from mice injected with MP *TRAC-*CAR T cells than Control *TRAC-*CAR T cells (Figure 5B), indicating improved T cell persistence *in vivo* for MP products. Among transgene^+^/TCR^−^ cells we found no significant differences in CD8 or CD4 expression with nearly 90% of cells being cytotoxic T lymphocytes across conditions (Figure S8).

We assessed splenic lymphocytes according to T cell differentiation and exhaustion using six markers: CD45RA, CD45RO, CD62L, CCR7, LAG3, and TIGIT. MP or Control *TRAC-*CAR T cells separated from each other in a reduced two-dimensional Uniform Manifold Approximation and Projection (UMAP) space (Figure 5C) while No CAR Control cells spanned the entire space. The vertical UMAP2 axis separated lymphocytes by differentiation status, with central memory (T_CM_) (CD45RO^+^/CD45RA^−^) and effector (T_EFF_) (CD45RA^+^/CD45RO^−^) T cells on opposing ends of the axis (Figures 5C and 5D). This differentiation *in vivo* is expected, as T_SCM_ will differentiate after antigen exposure.^53^^,^^54^ T_CM_ cells showed equal CD62L, less LAG3 and CCR7 but more TIGIT expression than T_EFF_ (Figure S9). Higher percentages of T_CM_ were found in the spleens of mice treated with MP *TRAC-*CAR T cells than with Control *TRAC*-CAR T cells (MP: 28% [16], Control: 5% [4], No CAR Ctrl: 18% [21], p [MP versus Control] = 0.0025) (Figures 5D and 5E), indicating improved central memory MP *TRAC-*CAR T cell persistence *in vivo*. In contrast, there were more T_EFF_ cells in spleens from mice treated with Control *TRAC*-CAR T cells (MP: 35% [18], Control: 83% [7], No CAR Ctrl: 54% [23], p [MP versus Control] < 0.001) (Figures 5D and 5E).

We also serially stimulated MP or Control *TRAC-*CAR T cells with CHLA-20 cells for 20 days to measure *in vitro* differentiation in response to chronic antigen stimulation. Spectral flow cytometry analysis revealed that MP *TRAC*-CAR T cells had increased persistence of naive T_SCM_ (MP: 52% [18], Control: 35% [14], p = 0.006) and “transitional” (MP: 75% [6], Control: 63% [12], p = 0.045) populations relative to Control *TRAC-*CAR T cells. Both groups also separated in a reduced two-dimensional t-SNE space with naive T cells clustering closer to MP *TRAC-*CAR T cells (Figure S10). Overall, these data indicate that MP *TRAC*-CAR T cells show better persistence *in vivo* and *in vitro*, with enrichment of T_CM_ cells noted in the spleen.

## Discussion

We improved upon the manufacturing process of an anti-GD2 *TRAC-*CAR T cell^41^ by adapting it for higher scales and enriching for desirable stem cell/central memory and metabolic phenotypes. Via priming T cells in medium with lower concentration of glucose, glutamine, and potentially other key nutrients, we subsequently reprogrammed T cell metabolism pre- and post-EP during *ex vivo* expansion. Our process used GMP-compatible reagents and can accommodate the EP of 100 million T cells in a “single-shot” cartridge and up to 25 billion cells in a “multi-shot” cartridge.^55^ We used CRISPR-Cas9 to insert a 3–4 kb transgene at the *TRAC* locus via HDR, a process that is more scalable than PCR-generated HDR templates due to the facile Nanoplasmid vector amplification within recombinant bacteria.^56^^,^^57^ Priming T cells with TexMACS supplemented with IL-7/IL-15 pre-EP to briefly slow glycolysis and proliferation followed by expansion in Immunocult XF with IL-7/IL-15 post-EP produced a final MP *TRAC*-CAR T product enriched for stem cell memory T cells with a unique metabolic profile that showed comparable potency with *TRAC*-CAR T cells generated under a single media source, but better persistence and higher enrichment of central memory T cells *in vivo*, an ongoing goal of the field of CAR T cell therapy for solid tumors.

Compared with Control cells, MP *TRAC*-CAR T cells had higher populations of CD62L^+^/CCR7^+^ cells, lower lactate production, glucose consumption, and ECAR post-manufacturing, making them distinctly more T_SCM_ like^13^^,^^22^^,^^49^ (Figures 2C, 2F, and 3A). These cells also had lower lactate production per mole of glucose consumed indicating a shift away from glycolysis (Figure 2C). In addition, MP *TRAC*-CAR T cells had higher mitochondrial membrane potential and mass when normalized to cell size (Figure 3B), which is often seen in memory cells as they switch to OXPHOS and fatty acid oxidation metabolism.^47^ Surprisingly, these cells also had lower rates of oxygen consumption and a lower OCR/ECAR (Figure 3A): this finding is consistent with another study on the effects of intermittent fasting *in vivo* in murine bone-marrow T cells where similarly low ECAR and OCR were observed along with reduced mTOR activity.^58^^,^^59^ The reduced OCR and ECAR in MP *TRAC-*CAR T cells could be due to reduced mTOR signaling, considering that mTOR is upregulated in effector T cells,^60^ and MP *TRAC-*CAR T cells were less cytotoxic than Control *TRAC*-CAR T cells *in vitro* (Figure 4), but further studies of the role of mTOR within MP *TRAC*-CAR T cells are needed.

The lower OCR, ECAR, and glucose consumption in MP *TRAC*-CAR T cells may have also reduced granzyme production, mediators of cytotoxic activity, especially given the lower granularity (side scatter by flow cytometry) in MP *TRAC*-CAR T cells. However, this reduced granularity *ex vivo* is overcome *in vivo* as both culture conditions produced *TRAC-*CAR T cells that can successfully suppress tumor growth in a xenograft mouse model (Figure S6). An advantage of MP *TRAC*-CAR T cells was the generation of more central memory cells compared with Control *TRAC*-CAR T cells *in vivo* (Figure 5), with better persistence in the periphery (spleen). These cells also maintained a more naive phenotype in *in vitro* serial stimulation assays, indicating delayed differentiation in MP *TRAC-*CAR T cells when chronically activated (Figure S10). Given this known limitation for retrovirally transduced anti-GD2 CAR T cells generated with standard biomanufacturing techniques to treat neuroblastoma,^5^ clinical trials with virus-free *TRAC*-CAR T cells with an MP biomanufacturing process will be needed to determine if enhanced persistence and central memory can be achieved in patients.

Our MP process demonstrates how modulating medium composition and cytokines of *TRAC-*CAR T cells pre- and post-EP influences CAR T cell phenotype, metabolism, and persistence post-infusion. While our study used *TRAC-*CAR T cells, there were no significant differences in CD62L/CCR7 expression based on CAR or TCR expression in our product. In addition, primary T cells undergoing MP have similar drops in lactate production and glucose consumption compared with control cells (Figure S11), suggesting the benefits of metabolic are not dependent on CAR or TCR expression and may extend to other modalities. While many studies have tuned culture conditions of viral CAR T cells to increase metabolic fitness, T_SCM_ properties, and potency,^16^^,^^17^^,^^25^^,^^27^^,^^29^ it is unclear if these benefit *TRAC-*CAR T cells, already having been shown to have more potent memory phenotypes.^41^ We and others have shown the importance of restricting glycolysis only during activation as expanding *TRAC-*CAR T cells in TexMACs ablates the metabolic phenotype adopted by MP *TRAC-*CAR T cells (Figure S2) and produces a lower cell yield (Figure S3).^42^ Activating T cells in TexMACs rewires their metabolism away from glycolysis (Figure S1) and it may be possible that factors in Immunocult XF can uniquely enforce this phenotype post-EP. Higher glutamine content in Immunocult-XF compared with TexMACs^17^ may create a glutamine deprivation during activation, which has been shown to increase metabolic fitness of therapeutic T cells.^25^^,^^26^ Lower glutamine during expansion in TexMACs may force T cells to take up more glucose to compensate and lower cell yield. In addition, while the medium choice during *TRAC-*CAR T cell culture seems to impact metabolism more than the choice of cytokines, priming anti-GD2 *TRAC-*CAR T cells with IL-2 could produce similar stem cell memory phenotypes with enhanced *in vivo* performance.^42^

Additional strategies will need to be explored to determine if these effects can be enhanced even further. For example, treating T cells during activation with N-acetylcysteine, a well-known antioxidant, can promote expression of stem cell memory markers and lower glycolysis.^29^ T cell metabolism could be manipulated using genetic engineering,^38^ as *PRODH2* overexpression can shift cells to an OXPHOS-based metabolism with more active mitochondria and an increased percentage of CD45RA^+^/CD62L^−^ T cells post-tumor challenge.^38^ Transient glucose restriction and treatment of T cells with the glutamine inhibitor DON have also both been shown to increase T_SCM_ surface marker expression and lower glycolysis. Advances to *TRAC-*CAR T cell biomanufacturing has the capacity to produce cell therapies with favorable biologic characteristics that could potentially improve CAR T cell responses against solid tumors.

## Materials and Methods

### Cell lines

GD2^+^ human neuroblastoma CHLA-20 cells were gifted by Dr. Mario Otto (University of Wisconsin-Madison). These cells were cultured in Dulbecco’s modified Eagle’s medium (DMEM) supplemented with 10% fetal bovine serum (FBS) (Gibco, Thermo Fisher Scientific, Waltham, MA) and 1% penicillin-streptomycin (P/S) (Gibco, Thermo Fisher Scientific). AkaLucGFP CHLA-20 cells were created through viral transduction by Dr. James Thomson (Morgridge Institute for Research). In short, Phoenix cells (ATCC, Manassas, VA) were grown in DMEM with 10% FBS and 1% P/S and selected with 1 μg/mL of diphtheria toxin (Cayman Biologics, Ann Arbor, MI) and 300 μg/mL hygromycin (Thermo Fisher Scientific). Selection for transgene-positive cells was confirmed by flow cytometry for mouse Lyt2 expression as a reporter gene (BioLegend, San Diego, CA). 3T3 cells were grown in DMEM with 10% FBS and 1% P/S. Cell authentication was performed using short tandem repeat analysis (Idexx BioAnalytics, Westbrook, ME) and as per ATCC guidelines using cell morphology, growth curves, and *Mycoplasma* testing within 6 months using the MycoStrip Mycoplasma Detection Kit (Invitrogen). Cell lines were maintained in culture at 37°C in 5% CO_2_.

### Plasmid constructs

A GD2-CAR plasmid construct encoding a 2A.14G2A-CD28-OX40-CD3ζ CAR gifted by Malcolm Brenner (Baylor College of Medicine) was synthesized and the sequence verified (GenScript, Piscataway, NJ). A separate No CAR Control (mCherry) construct was contained in an H2B-mCherry sequence in place of the GD2-CAR and designed, synthesized, and sequenced the same as the GD2-CAR plasmid (GenScript). Both transgenes were flanked by 500 bp homology arms and cloned into a pUC57 backbone, grown in 5-alpha competent *Escherichia coli* (NEB, Ipswich, MA) and purified using the PureYield MidiPrep System (Promega, Madison, WI).^41^

### Sanger sequencing

The GD2-CAR and No CAR Control templates had their sequences verified via Sanger sequencing at the UW-Biotechnology Center (Madison, WI). In brief, each construct was separated into 20 μL aliquots with appropriate primers for sequencing found in Table S3. PCR was performed, the amplicons separated by gel electrophoresis, and peaks analyzed for sequence identity.

### Nanoplasmid production

Linear dsDNA templates were made via PCR and amplification was done using the GD2-CAR or No CAR Control plasmid as a template using Q5 Hot Start Polymerase (cat. no. M0494S, NEB) in 50 μL reaction volumes. The cycling parameters were 98°C for 10 s, 65°C for 20 s, and 72°C for 90 s, for a total of 35 cycles. These reactions were then pooled into 600 μL reactions for solid-phase reversible immobilization (SPRI) cleanup (1×) using AMPure XP (cat. no. B23318, Beckman-Coulter, Brea, CA) beads according to the manufacturer’s instructions and eluted at 2 mg/mL in DNAase-free water. The linear products were shipped to Aldevron (Fargo, ND) where they were blunt cloned into a Nanoplasmid backbone consisting of two components: R6K origin of replication and an anti-levansucrase RNA sequence that enables antibiotic free selection. The RNA-OUT platform prevents the expression of SacB which prevents toxicity from Levansucrase as well as transgene silencing after genomic insertion.^57^^,^^61^ Nanoplasmid DNA was manufactured on-site at Aldevron and resuspended in DNAase-free water to 2 mg/mL. Nanoplasmid sequences can be found in Table S2. Primer sequences are shown in Table S3.

### Nanoplasmid linearization by restriction digest

To linearize Nanoplasmid for use as a dsDNA HDR template, a restriction digest of the Nanoplasmid constructs using SSPI-HF (cat. no. R3132S, NEB, Ipswich, MA) was performed. Four restriction digest batch reactions in 1.5 mL Eppendorf tubes (50 μL Nanoplasmid, 125 μL CutSmart buffer, 25 μL SSPI-HF enzyme, and 1050 μL DNAase-free water for 1,250 μL total) were aliquoted (50 μL into PCR tubes for a total of 96 reactions). These were incubated in a thermocycler at 37°C for 15–60 min and heat inactivated at 65°C for 20 min according to the manufacturer’s instructions. Gel electrophoresis was then performed on the finished product to assess if proper cutting took place, followed by SPRI cleanups to purify and concentrate the material to 2 mg/mL. PCR reactions were pooled into eight 1.5 mL Eppendorf tubes (600 μL) with an equal volume of solid-phase reversible immobilization (SPRI) (Beckman-Coulter) beads that were incubated for 5 min at room temperature. The product was washed twice with 70% ethanol and eluted in 75 μL of DNase-free water and pooled into one tube (600 μL). This product was subject to a second round of cleanups and eluted in 30 μL of water. DNA was quantified using the NanoDrop2000 Qubit dsDNA Broad Range (BR) Assay (ThermoFisher Scientific, Waltham, MA) and diluted to 2 mg/mL according to Qubit measurements.

### Isolation of T cells from peripheral blood

Peripheral blood was drawn from healthy donors using an IRB-approved protocol (UW-Madison 2018-0103). Blood was collected into lithium heparin-coated vacutainer tubes and transferred to 50 mL conical tubes. CD3^+^ primary human T cells were isolated by negative selection as per the manufacturer’s instructions (cat. nos. 15021 and 15061, RosetteSep Human T cell Enrichment Cocktail, STEMCELL Technologies, Vancouver, Canada). T cell pellets were resuspended in dilution medium and counted using a hemocytometer with 0.4% trypan blue viability stain (Thermo Fisher Scientific). Cells were then resuspended at 1 million/mL in either Immunocult-XF T cell Expansion Medium (cat. no. 10981, STEMCELL Technologies) or TEXMACs Cell Culture Medium (cat. no. 130-097-196, Miltenyi Biotec, Bergisch Gladbach, Germany). T cell cultures were supplemented with 200 U/mL IL-2 (cat. no. 200-02, PeproTech, Cranbury, NJ) or 10 ng/mL of IL-7 (cat. no. 207-IL-005/CF, BioTechne, Minneapolis, MN) and 10 ng/mL of IL-15 (cat. no. 247-ILB-005/CF, BioTechne) and stimulated with Immunocult Human CD3/CD28/CD2 T cell Activator (25 μL for each mL of culture, cat. nos. 10990 and 10970, STEMCELL) for 48 h or T cell TransAct (10 μL for each mL of culture, cat. no. 130-111-160, Miltenyi Biotec) for 72 h, respectively.

### Isolation of T cells from LRS cones

Leukocyte reduction system (LRS) cones (Versiti Blood Bank, Milwaukee, WI) were purchased as an alternative to drawing from healthy donors. In brief, red blood cell (RBC)-depleted peripheral blood was flushed out of the cone with a blunt gauge needle and dilution medium (2% FBS in dPBS). The solution was diluted 1:1 with dilution medium and 30 mL was carefully layered on top of 15 mL of Lymphoprep (cat. no. 07811, STEMCELL Technologies) solution. Tubes were centrifuged for 1,200 × *g* × 20 min with the brake off. The white monolayer containing leukocytes was then gently pipetted into a 15 mL conical tube, which was spun at 300 × *g* × 5 min and washed twice. T cells were then positively selected using an EasySep T cell kit (cat. no. 17951, STEMCELL Technologies) and an EasySep Magnet (STEMCELL Technologies) as per the manufacturer’s protocol. T cell supernatant was collected from the magnet and the cells were then counted, resuspended in culture medium, and activated as above.

### T cell electroporation on the Lonza 4D Nucleofector

Following T cell activation, RNPs and DNA were electroporated into T cells in 16 well EP cuvettes on a 4D Nucleofector X unit (cat. no. V4XP-3032, Lonza, Walkersville, VA) using pulse code EH-115. One million cells were electroporated per well in the cuvette. Per reaction, a single-guide RNA (Integrated DNA Technologies, Coralville, IA) specific for the TRAC locus (2 μL of 100 μM, IDT) was incubated with SpCas9 (0.8 μL of 10 mg/mL, cat. no. 9212–0.25MG, Aldevron, Madison, WI) and poly-L-glutamic acid (PGA [15,000–50,000 kDa], 1.6 μL of 10 mg/mL solution in DNase-free water, cat. no. 26247-79-0, Millipore Sigma, Burlington, MA) for 15 min at 37°C to form the RNP complex. During incubation, T cells were centrifuged for 300 × *g* for 5 min and counted on the Countess II FL Automated Cell Counter (Thermo Fisher Scientific) with 0.4% trypan blue viability stain. One million cells per reaction were then aliquoted and spun for 90 × *g* for 10 min. Following RNP incubation, linearized dsDNA HDR templates were added to the mixtures (2 μL) in PCR tubes and incubated for at least 5 min. Cells were then resuspended in 17.6 μL of P3 buffer (Lonza) and transferred to PCR tubes containing the RNP:DNA mixtures. Contents were then transferred to cuvettes (total volume 24 μL) and electroporated. Immediately following EP, 80 μL of Immunocult XF (STEMCELL Technologies) or TexMACS medium (Miltenyi Biotec) with *no cytokines* was added to each reaction, which were then rested for 30 min at 37°C. Cells were then moved to a flat-bottom 96 well plate containing 160 μL of medium supplemented with 500 U/mL IL-2 (PeproTech, Cranbury, NJ) or 10 ng/mL of IL-7 (BioTechne) and 10 ng/mL of IL-15 (BioTechne). Cells were cultured for 24 h and then transferred to 12-well plates with 1 mL of medium and incubated at 37°C for 48 h. The *TRAC* guide sequence can be found in Table S3.

### T cell culture

MP or Control *TRAC-*CAR T cells were cultured in TexMACS or Immunocult XF medium supplemented with IL-7/IL-15 (10 ng/mL) or IL-2 (200 U/mL) at 1 million cells/mL, respectively, for the first 3 days. After EP, both MP and Control *TRAC-*CAR T cells were cultured in Immunocult XF for 7 days with IL-7/IL-15 (10 ng/mL) or IL-2 (500 U/mL). Every 2 days, cells were centrifuged for 300 × *g* for 5 min and counted on the Countess II FL Automated Cell Counter (ThermoFisher, Waltham, MA) with 0.4% trypan blue viability stain. Cells were then resuspended in culture medium at 1 million cells/mL and the process was repeated on days 5 and 7 post-EP.

### Scaled-up GMP-compatible T cell manufacturing

For GMP-compatible experiments, T cells were isolated and activated and cultured with either TransAct in TexMACS supplemented with IL-7 (10 ng/mL) or IL-15 (10 ng/mL) or with Immunocult XF activator and medium supplemented with 200 U/mL IL-2. Following activation, 20–50 million cells were electroporated on the CTS Xenon Electroporation System (Thermo Fisher Scientific). TRAC sgRNA (1 μL/1e6 cells) (IDT, Coralville, IA) and SpCas9 (0.8 μL/1e6 cells) (Aldevron, Madison, WI) were mixed and incubated for 15 min at 37°C. Following incubation, linearized Nanoplasmid template 1 μL/1e6 cells) was then added to the RNP mixtures. Cells were spun, counted, spun again for 5 min at 300 × *g*, and resuspended in enough Genome Editing Buffer (cat. no. A4998001, Thermo Fisher) for a final volume of 1 mL when combined with the RNP:DNA mixture. Cells, DNA, and RNPs were then added together, loaded into a single-shot cuvette (cat. no. A50305, Thermo Fisher), and electroporated on the Xenon unit at 1,720 V with a pulse width of 20 ms. Cells were then transferred to a T-25 flask with 4 mL of Immunocult XF medium containing no additives. After 30 min of rest at 37°C, Immunocult XF medium containing either IL-7 (10 ng/mL) with IL-15 (10 ng/mL) or 500 U/mL IL-2 was added to the T-75 flasks to bring the final concentration to 4 million cells/mL. After 24 h, cells were transferred to a G-Rex 6M plate (10–e6 per well) (cat. no. P/*N* 80660M, Wilson Wolf, New Brighton, MN) and the final volume brought to 100 mL per well. Cells were then cultured at 37°C for 6 days, after which 75 mL of medium was aspirated, cells were collected, spun for 300 × *g* for 5 min, and counted for use in endpoint assays.

### Flow cytometry analysis

CAR was detected using a 1A7 anti-14G2A antibody (National Cancer Institute, Biological Resources Branch), conjugated to APC using a Lightning Link APC Antibody Labeling kit (cat. no. 705-0010, Novus Biologicals). TCR was detected using an anti-human TCR α/β antibody conjugate to BV421 (BioLegend). Flow cytometry to assess CAR and TCR positivity was performed on day 8 of manufacturing on an Attune NxT flow cytometer (Thermo Fisher Scientific). Immunophenotyping of cells was performed on day 10 of manufacturing using a spectral immunophenotyping panel on an Aurora spectral cytometer (Cytek, Fremont, CA). In brief, cells were plated in a 96-well round bottom plate (100k for CAR/TCR and 250k for spectral immunophenotyping), washed with 200 μL of PBS, and spun at 1,200 × *g* for 1 min, twice. Cells were then stained for viability with either GhostRed 780 (cat. no. 50-105-2988, Tonbo Biosciences) or Live-Dead Blue (cat. no. L23105, Thermo Fisher Scientific). For CAR/TCR staining, 1 μL of Ghostred 780 was added to 10 mL of PBS to make a stock solution, 100 μL of stock solution was added to each sample and incubated for 30 min in the dark. For spectral flow staining, Live-Dead Blue stain was resuspended in 50 μL of DMSO, 1 μL added per 1 mL PBS to make a stock solution, and 200 μL of stock solution was added to each sample and incubated for 30 min in the dark. After viability staining, samples were washed twice and blocked for 30 min with 50 μL FACS buffer (0.5% BSA in PBS) with TruStain FcX solution (0.5 μL/sample) (cat. no. 422301, BioLegend). Antibodies were then added to 100 μL of BD Brilliant Stain Buffer (cat. no. 659611, BD Biosciences, Franklin Lakes, NJ) at the optimized amounts found in Table S4 and incubated for 1 h. Cells were then washed, resuspended in 200 or 75 μL of FACS buffer, and analyzed on the Attune or Aurora, respectively. For spectral immunophenotyping, we used CD4, CD8, TCR, and CAR positivity to define populations and for all markers cells were gated by relative size, shape, singlets, viability, TCR negativity, and CAR transgene positivity to find an analyzable population of viable CAR T cells. All antibodies are listed in Table S4.

### *In vitro* cytotoxicity assay on IncuCyte

A total of 5,000 AkaLUC-GFP CHLA-20 cells was seeded in triplicate on 96-well plates and incubated for 24 h at 37°C. Twenty-four hours later 50,000, 25,000, or 10,000 CAR^+^ T cells from day 10 of manufacturing were added to each well for effector:target ratios of 5:1, 2.5:1, or 1:1. The plate was centrifuged for 5 min at 100 × *g* and then placed in the IncuCyte S3 Live-Cell Analysis System (Sartorius, Gӧttingen, Germany) and stored at 37°C, 5% CO_2_. Images were taken every 3 h for 48 h. Green object count was used to calculate the number of cancer cells in each well and fluorescent images were analyzed with IncuCyte Base Analysis Software.

### *In vivo* infusion into mice with human xenografts

All animal experiments were approved by the University of Wisconsin-Madison Animal Care and Use Committee (ACUC protocol M005915). Male and female NOD-SCID-γc^−/−^ (NSG) mice (9–25 weeks old; Jackson Laboratory, Bar Harbor, ME) were subcutaneously injected with 10 million AkaLUC-GFP CHLA-20 GD2^+^ human neuroblastoma cells in the flank to establish tumors. After 6 days, tumor size was verified using bioluminescence measurements on the *In Vivo* Imaging System (IVIS) (PerkinElmer, Waltham, MA) and 3 million CAR^+^ T cells from day 10 of manufacturing were injected into the tail vein of each mouse. Mice were imaged on the IVIS every 3–4 days after being sedated with isoflurane and intraperitoneal injections of ∼120 mg/kg D-luciferin (GoldBio, St. Louis, MO). Mice were injected with 100,000 IU of human IL-2 (National Cancer Institute, Biological Resources Branch) subcutaneously on day 0 and following imaging. To quantify the total flux in images, a region of interest was drawn around established tumors on day 0 and calculated by Living Image Software (PerkinElmer; total flux = radiance (photons/s) in each pixel integrated over ROI area (cm^2^) × 4π). The minimum flux value was subtracted from each image to normalize for background signal.

### Flow cytometry of splenocytes

Spleens were removed, mechanically dissociated, and filtered using a Corning 70 μM cell strainer. Suspensions were centrifuged for 10 min at 300 × *g* and digested with ACK lysing buffer (Lonza). The cells were then washed with PBS, centrifuged for 10 min at 300 × *g*, and resuspended in 1 mL of PBS. Cells were counted using trypan blue exclusion on the Countess II FL Automated Cell Counter. A total of 1 × 10^6^ total cells was then added to 96-well round bottom plates and stained for a spectral immunophenotyping panel for analysis on an Aurora spectral cytometer (Cytek). In brief, samples were washed with PBS, stained with Live-Dead Blue for 30 min, blocked with FACS buffer and Trustain FcX, and incubated with the spectral immunophenotyping panel overnight. For spectral immunophenotyping, we used CD4, CD8, TCR, and CAR positivity to define populations and for all markers cells were gated by relative size, shape, singlets, viability, CD5 positivity, CD45 positivity, TCR negativity, and CAR transgene positivity to find an analyzable population of viable CAR T cells. All antibodies and amounts are listed in Table S4.

### *In vitro* serial stimulation and flow cytometry analysis of lymphocytes

We added 120,000 CHLA-20 cells in duplicate to 6-well plates. We then seeded 120,000 CAR^+^ T cells from day 10 of manufacturing and incubated cells at 37°C, 5% CO_2_. A total of 240,000 CHLA-20 cells was then added every 2–3 days for up to 20 days when the wells were harvested and stained for flow spectral cytometry. In brief, CHLA-20/CAR T cell samples were washed with PBS, stained with Live-Dead Blue for 60 min, blocked with FACS buffer and Trustain FcX, and incubated with the spectral immunophenotyping panel overnight. For spectral immunophenotyping, we used TCR negativity to define populations and all markers cells were gated by relative size, shape, singlets, viability, CD5 positivity, CD45 positivity, and TCR negativity to find an analyzable population of viable T cells. All antibodies and amounts are listed in Table S4.

### Cytokine analysis

The supernatant of CAR T and cancer co-culture systems was measured for their expression of cytokines using the LegendPlex Human Essential Immune Response Panel (cat. no. 740930, BioLegend) (IL-4, IL-2, CXCL10 [IP-10], IL-1β, TNF-α, CCL2 [MCP-1], IL-17A, IL-6, IL-10, IFN-γ, IL-12p70, CXCL8 [IL-8], TGF-β1 [free active form]). In brief, 50,000 AkaLUC-GFP CHLA-20 neuroblastoma cells were plated in 24-well plates with the addition of 250,000 CAR T cells 24 h later. Suspension cells were harvested 24 h later, spun for 5 min at 300 × *g*, cells counted, the supernatant flash frozen in liquid nitrogen, and stored at −20 C. Medium samples were thawed and the manufacturer’s protocol for bead staining and analysis on an Attune NxT Cytometer (Thermo Fisher Scientific ) was followed. In brief, standards for cytokines were prepared using 1:4 serial dilutions and co-culture supernatant added directly to a 96-well V-bottom plate. Mixed beads were added to each well and incubated on a plate mixer for 2 h. The plate was then washed twice, detection antibody added to each well, incubated for 1 h, and Streptavidin A (SA)-PE was added to each well. The plate was incubated for 30 min, washed twice, and analyzed on the Attune Cytometer. Data were exported to the LEGENDplex Data Analysis Software Suite where cytokine concentration was calculated. The data were exported to Excel, and cytokine concentration was normalized to the total number of T cells in co-culture.

### Mitochondrial staining

The mitochondrial mass and membrane potential of T cells was measured by performing flow cytometry on cells stained with MitoTracker Green (cat. no. M7514, Thermo Fisher Scientific) and tetramethylrhodamine methyl ester perchlorate (TMRE) (Thermo Fisher Scientific) dyes. In brief, a stock solution of PBS containing 0.08 μL/mL of MitoTracker Green and 0.1 μL/mL of a 10 μM TMRE dye stock was created (200 μL per sample of 250,000 T cells). Cells were stained for 20 min at 37°C, washed, and resuspended in 75 μL of FACS buffer (0.5% BSA in PBS).

### Metabolite analysis

Medium samples were taken from CAR T cell cultures on day 10 of manufacturing and frozen at −20°C for future analysis. The Glucose-Glo and Lactate-Glo kits (cat. nos. J6021 and J5021, Promega, Madison, WI) were used to measure the apparent glucose and lactate concentrations in medium samples according to the manufacturer’s protocol. Raw luminescence data were converted to concentration using the metabolite standards.

### Extracellular flux assay (Seahorse assay)

The OCR and ECAR were measured following the manufacturer’s instructions for the Seahorse XF Cell Mito Stress Test Kit (Agilent, Madison, WI). In brief, 5 × 10^5^ T cells were resuspended in RPMI XF medium supplemented with 10 mM glucose and 2 mM glutamine and plated in a poly-L-lysine-coated XF96 plate. The T cell culture plate was centrifuged at 200 × *g* for 1 min (no brake) and checked under the microscope to ensure even adhesion of T cells. T cells were then kept in a non-CO_2_ incubator for at least 1 h before running the assay. The OCR and ECAR under basal conditions and in response to oligomycin (2.5 μM), fluorocarbonylcyanide-phenylhydrazone (1 μM), and rotenone/antimycin A (0.5 μM) were measured using an XF96 Extracellular Flux Analyzer (Seahorse Bioscience, Madison, WI). Data was exported into Excel (v.2311) using Agilent software and converted into graphs.

### Spectral flow cytometry data analysis

Analysis of spectral flow cytometry data was performed using Cytek’s SpectroFlo program, Single positive controls for each color were collected and analyzed in SpectroFlo for positive and negative populations. SpectroFlo’s unmixing algorithm was then used to compensate for spillover and autofluorescence of cells. Data were then exported to FlowJo (v.10.9.0) where samples were gated for non-debris, singlets, and live cells. TCR and CAR positivity were used to gate cell populations for *in vitro* samples and CD45, CD5, TCR, and CAR positivity for *in vivo* T cell samples. Median fluorescent intensity for each sample was calculated and input into Excel. Representative plots were generated in FlowJo using fluorescence minus one controls to set positive gates.

### Optical metabolic imaging

As described previously,^62^^,^^63^ 200,000 CAR T cells in 75 μL fresh medium were plated on a poly-D-lysine-coated 35 mm glass-bottom imaging dish (MatTek, Ashland, MA). Autofluorescence signals from NAD(P)H and FAD were imaged on a two-photon microscope (Ti-E, Nikon, Tokyo, Japan) at 750 nm excitation (440/80 nm emission) and 890 nm excitation (550/50 nm emission), respectively. Fluorescence decay was collected with time-correlated single-photon counting electronics (SPC 150, Becker & Hickl) and fitted to a two-component exponential decay in SPCImage (v.8.0, Becker and Hickl) to extract mean fluorescence lifetime (τ_m_) and fractional contribution of short- and long-lifetime components (α_1_ and α_2_). Single-cell nucleus and cytoplasm were manually segmented with a customCellProfiler pipeline and applied to the corresponding OMI images to quantify mean values of OMI parameters for each cell cytoplasm.

### Data analysis and software

All data analyses were performed in GraphPad Prism (v.10.0.2) and Microsoft Excel. Statistical tests were done in GraphPad Prism and indicated in the figure legends. Nanoplasmid sequences were designed in Benchling. FlowJo was used to analyze .fcs files exported from SpectroFlo and Attune NxT software. Representative flow plots were exported from FlowJo. UMAPs were created using the DownSample and UMAP_R plugins from to FlowJo to select for and cluster pooled data in a concatenated .fcs file. Figures were created and organized using Adobe Illustrator (v.28.0). A p value less than 0.05 was defined as significant. Cohen’s effect size was calculated by dividing the difference between treated and control group means and dividing by the pooled standard deviation of them.

## Data and code availability

Data are available upon request.

## Supplemental information

Data S1. Tables S2–S4

Document S2. Article plus supplemental information
