# Supplementary material for: Metabolic priming of GD2 TRAC-CAR T cells during manufacturing promotes memory phenotypes while enhancing persistence
Source: Mol Ther Methods Clin Dev. 2024 Apr 10;32(2):101249. doi: 10.1016/j.omtm.2024.101249 (PMC11063605; doi:10.1016/j.omtm.2024.101249)
Supplement: Document S1. Figures S1–S11 and Table S1 [file mmc1.pdf]

OMTM, Volume *32*

Supplemental information

Metabolic priming of GD2 *TRAC*-CAR T cells during manufacturing promotes memory phenotypes while enhancing persistence

Dan Cappabianca, Dan Pham, Matthew H. Forsberg, Madison Bugel, Anna Tommasi, Anthony Lauer, Jolanta Vidugiriene, Brookelyn Hrdlicka, Alexandria McHale, Quaovi H. Sodji, Melissa C. Skala, Christian M. Capitini, and Krishanu Saha

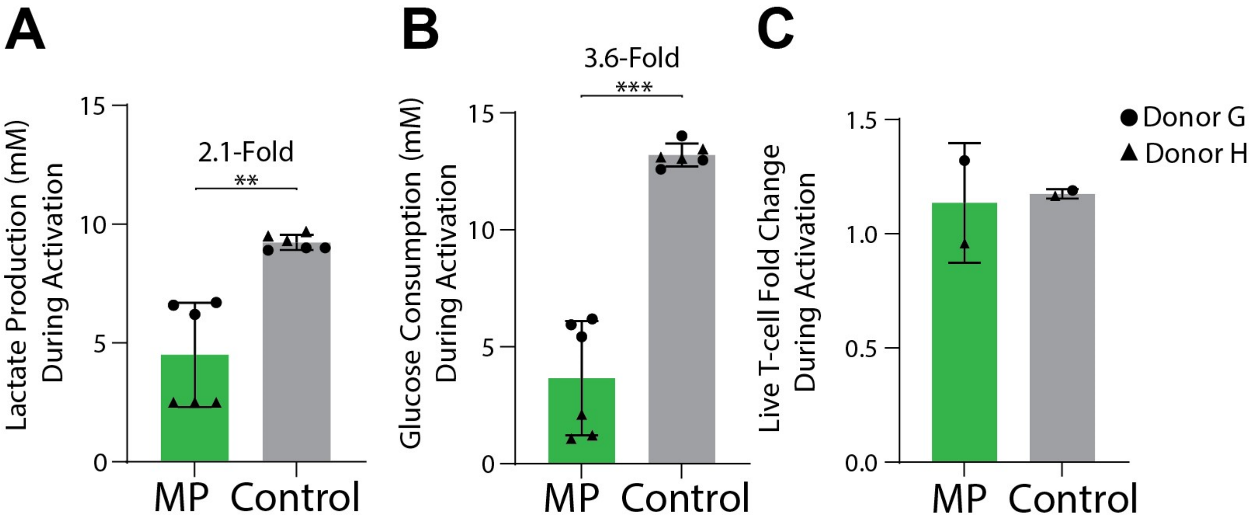

Figure S1. Effect of Activation Conditions on Lactate Production and Glucose Consumption. (A) Lactate production, (B) glucose consumption and (C) proliferation of MP or Control T cells during activation (MP – 72 hours; Control – 48 hours). 2 donors, (lactate/glucose) $N_{MP} = N_{Control} = 6$, (proliferation) $N_{MP} = N_{Control} = 2$. Error bars represent mean and standard deviation. Statistical significance was determined with a paired-test; **$p<0.01$; ***$p<0.001$.

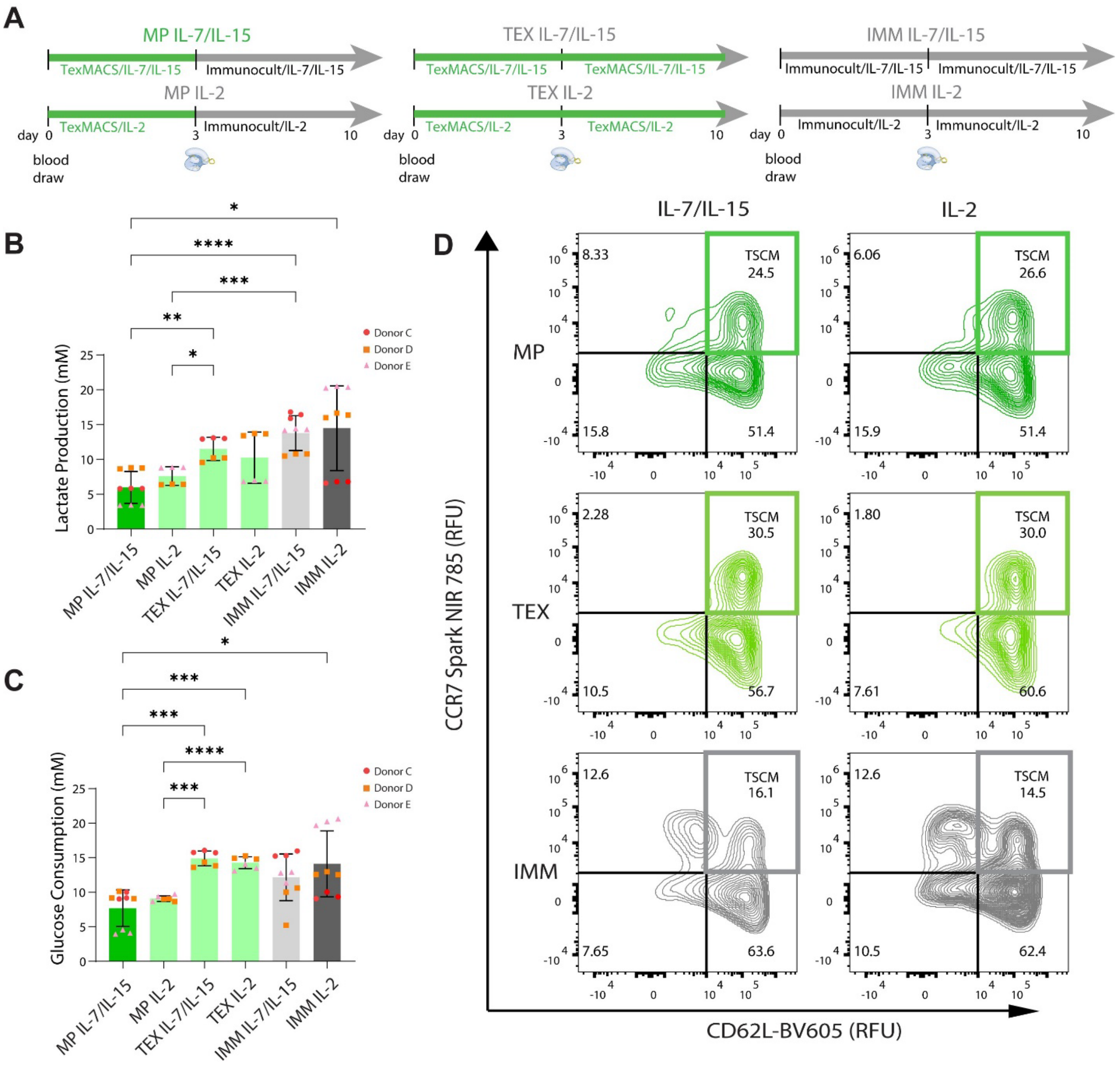

Figure S2. Stem Cell Memory Phenotypes of Differentially Cultured *TRAC*-CAR T cells. (A) CAR T cells were manufactured under six different media/cytokine conditions: 1). Metabolic priming with IL-7/IL-15 (MP), 2). metabolic priming with IL-2, 3). TexMACs with IL-7/IL-15, 4). TexMACs with IL-2, 5). Immunocult with IL-7/IL-15, and 6). Immunocult with IL-2 (Ctrl). Bar graphs of **(B)** lactate production (MP IL-7/I,-15: 6.0 mM (2.3), MP IL-2: 7.6 (1.3), TEX IL-7/IL-15: 11.5 (1.7), TEX IL-2: 10.3 (3.7), IMM IL-7/IL-15: 13.8 (2.5), IMM IL-2: 14.5 (6.1), *p* (IMM IL-2 vs MP IL-7/IL-15) = 0.0341, *p* (TEX IL-7/IL-15 vs MP IL-2) = 0.0147, *p* (TEX IL-7/IL-15 vs MP IL-7/IL-15) = 0.0017, *p* (IMM IL-7/IL-15 vs MP IL-2 or IL-7/IL-15) < 0.001. and **(C)** glucose consumption (MP IL-7/I,-15: 7.7 (2.6) , MP IL-2: 9.1 (0.4), TEX IL-7/IL-15: 14.9 (1.1), TEX IL-2: 14.3 (0.9), IMM IL-7/IL-15: 12.2 (3.4), IMM IL-2: 14.1 (4.8), *p* (IMM IL-2 vs MP IL-7/IL-15) = 0.046, *p* (TEX IL-2 or IL-7/IL-15 vs MP IL-2 or IL-7/IL-15) < 0.001 for all) from day 8 to 10 of manufacturing of differentially-cultured *TRAC*-CAR T cells. **(D)** Representative contour plots of the expression of CCR7 vs CD62L on Day 10 of manufacturing. 2-3 donors, $N_{MP, IL-7/IL-15} = 9$, $N_{MP, IL-2} = 6$, $N_{TEX, IL-7/IL-15} = 6$, $N_{TEX, IL-2} = 6$, $N_{IMM, IL-7/IL-15} = 9$, $N_{IMM, IL-2} = 9$. Error bars represent mean and standard deviation. Statistical significance was determined with Brown-Forsythe and Welch ANOVA tests using Dunnett's T3 test for multiple comparisons; *p<0.05; **p<0.01; ***p<0.001; ****p<0.0001.

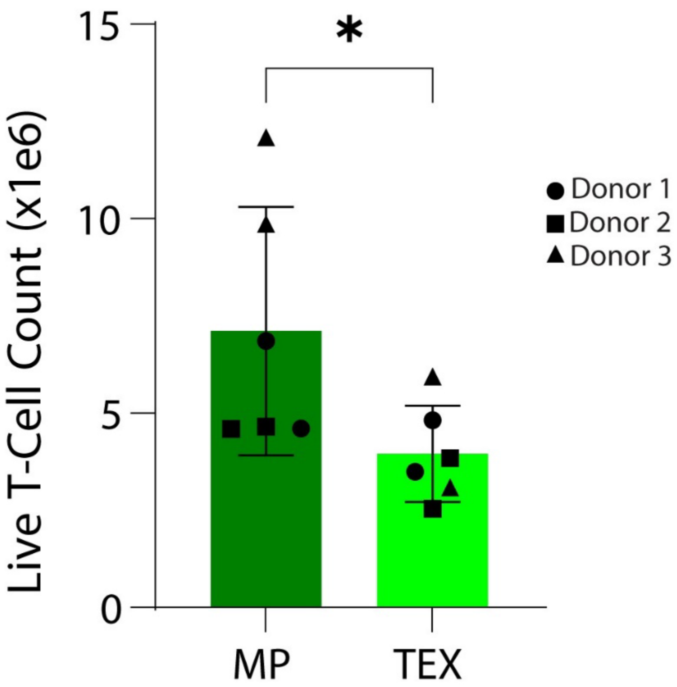

Figure S3. *TRAC*-CAR T Cell Expansion Under MP and TEX Cultures Conditions. *TRAC*-CAR T cells were manufactured with MP (metabolic priming) or TEX (TexMACs media only) culture, both supplemented with IL-7/IL-15. Bar graph of the live T-cell count on Day 5 post-EP of manufacturing (MP: 7.2e6 cells (3.2), TEX: 4.0e6 cells (1.2), $p = 0.041$). 3 donors, $N_{MP} = 6$, $N_{TEX} = 6$. Error bars represent mean and standard deviation. Statistical significance was determined with paired t-tests; *$p<0.05$.

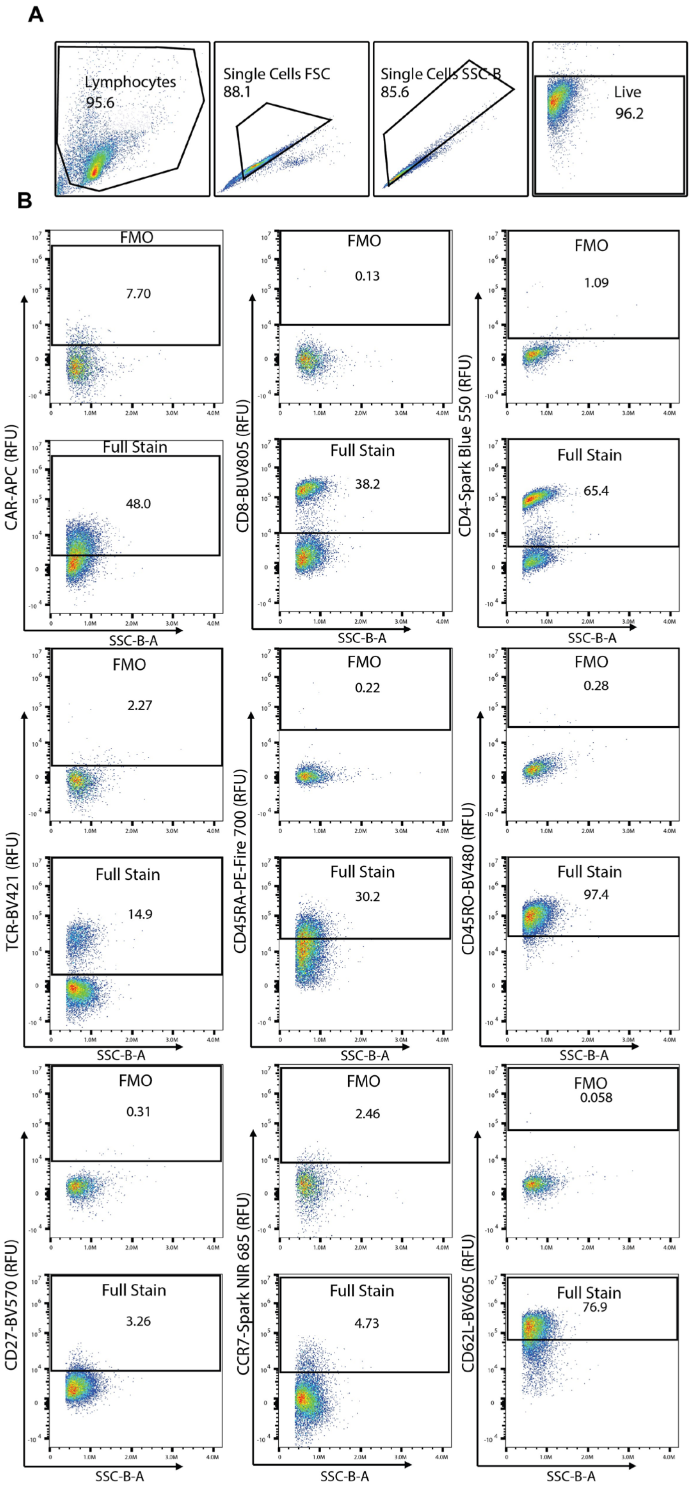

Figure S4. Post-Manufacturing CAR T Cell Gating Strategy. (A) Gating strategy for analysis of spectral immunophenotyping flow cytometry data of CAR T cells post-manufacturing. **(B)** FMO's and representative positive populations depicting positive and negative gates.

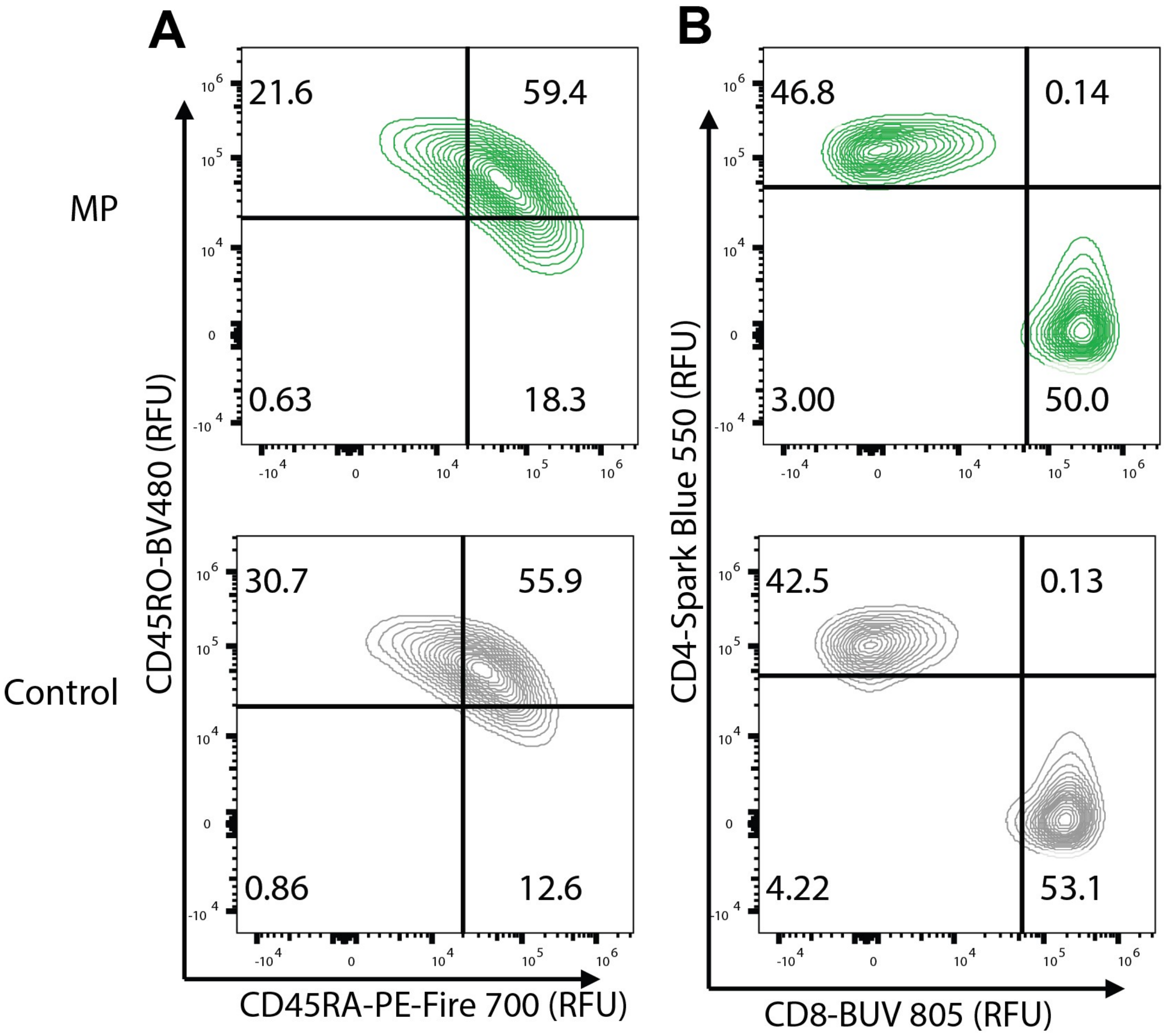

Figure S5. Expression of CD45RA/CD45RO and CD4/CD8 in MP *TRAC*-CAR T Cells at Scale. Representative contour plots for expression of (A) CD45RA/CD45RO and (B) CD4/CD8 MP or Control *TRAC*-CAR T cells (Donor F shown).

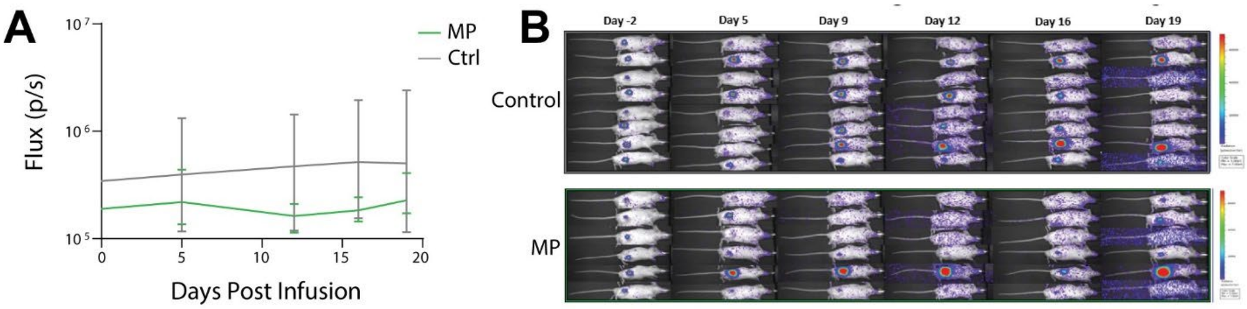

Figure S6. In Vivo Potency of MP and Control TRAC-CAR T cells. (A) The flux over time (luminescence from IVIS images) is shown for mice treated with MP or Control *TRAC*-CAR T cells. **(B)** IVIS images depict tumor growth over time for the same conditions. (2 donors, $N_{MP} = 6$, $N_{Control} = 8$). Error bars represent mean and standard deviation.

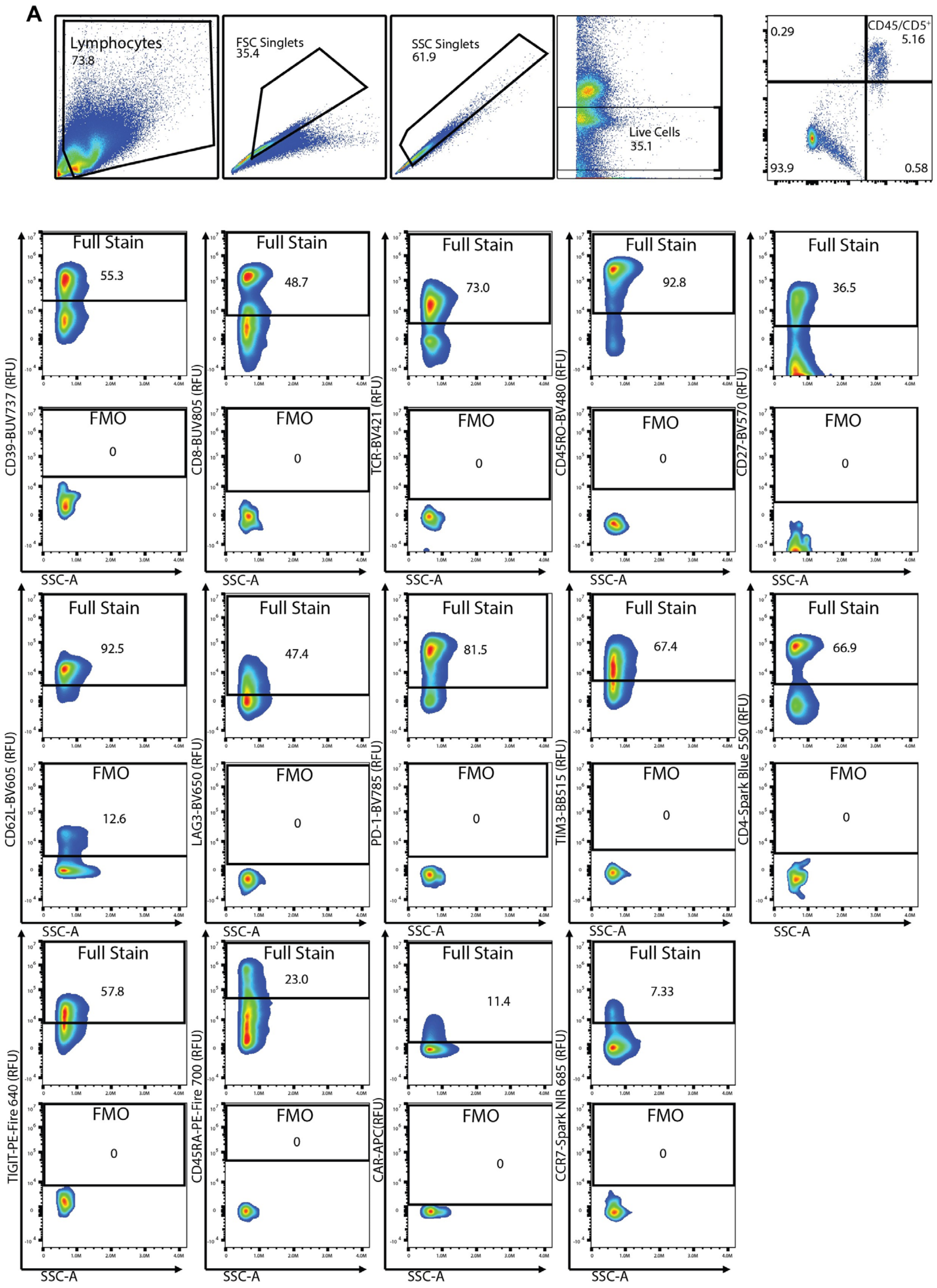

Figure S7 Gating Scheme for Analysis of Lymphocytes in Mouse Spleens. Gating strategy for analysis of live, CD5/CD45^{+} lymphocytes in isolated mouse spleens. **(B)** FMO's and representative positive populations depicting positive and negative gates.

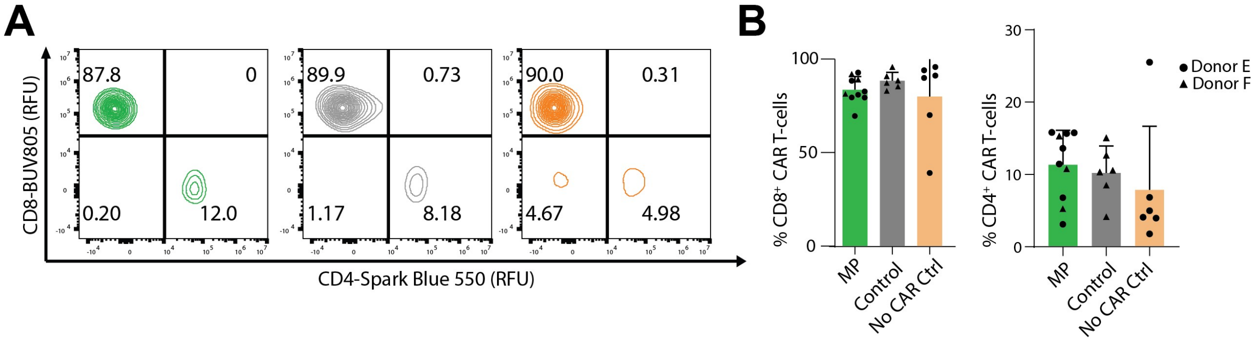

Figure S8. Expression of CD4 and CD8 Post In Vivo Tumor Challenge. (A) Representative contour plots for CD4/CD8 expression in CD5+/CD45+/TCR-/transgene+ lymphocytes isolated from mouse spleens (Green = MP, Donor E; Gray = Control, Donor F; Orange = No CAR Ctrl, Donor E). **(B)** Bar graphs for relative expression of CD4 and CD8 for MP or Control -CAR T cells and no CAR control T-cells. Samples with less than 20 CD5+/CD45+/TCR-/transgene+ events were excluded from analysis. 2 donors, $N_{MP} = 10$, $N_{Control} = 6$, $N_{NoCARCtrl} = 6$. Error bars represent mean and standard deviation.

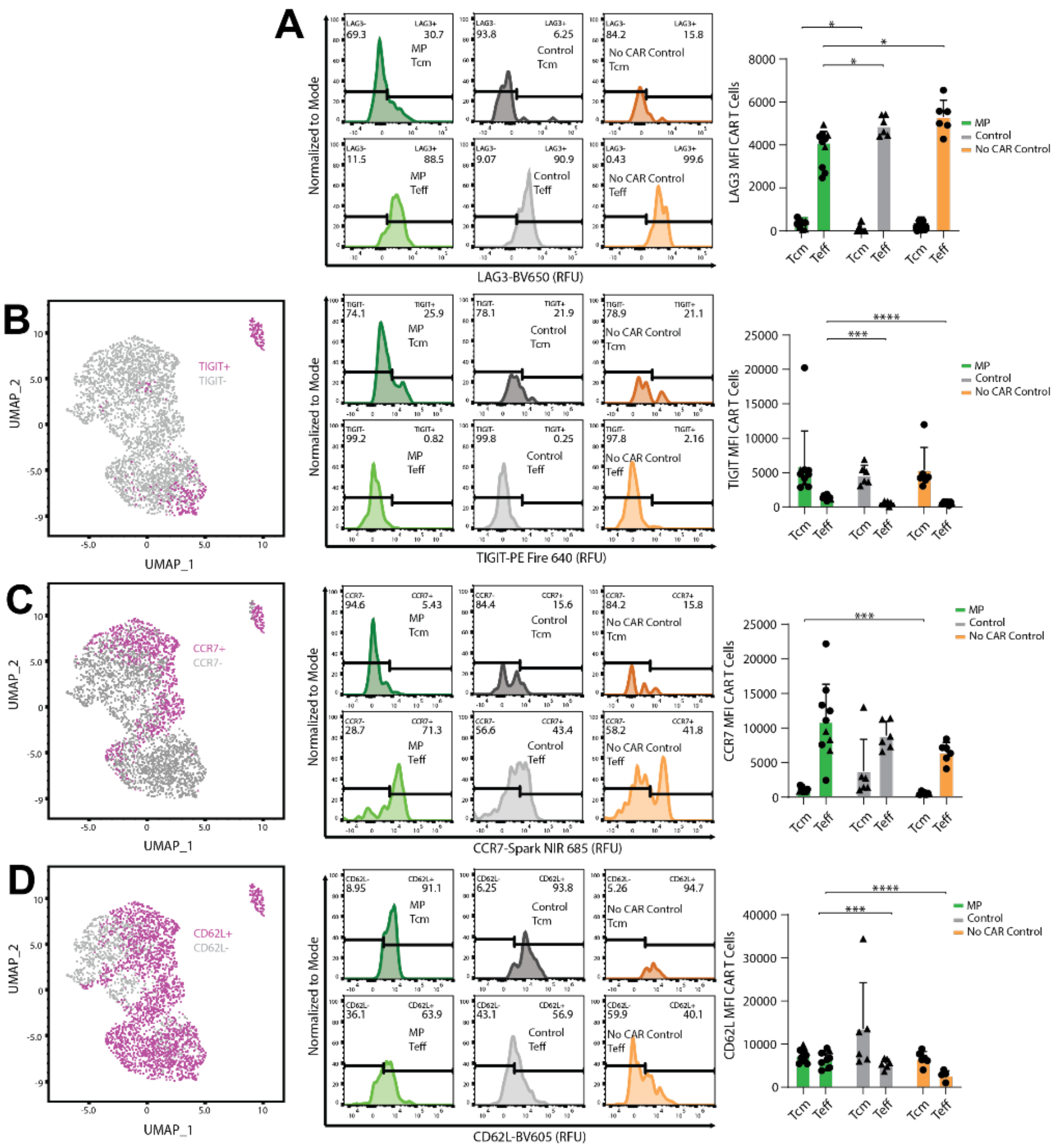

Figure S9. Expression of Exhaustion and Memory Markers in Splenic Lymphocytes. The expression of **(A)** LAG3 (Tcm: MP: 730 (342), Control: 115 (200), No CAR Control: 325 (325), p (MP vs Control) = 0.0014) (Teff: MP: 4117 (508), Control: 4895 (467), No CAR Control: 5311 (774), p (MP vs Control and MP vs No CAR Control) = 0.0278 and 0.0274), **(B)** TIGIT (Tcm: MP: 5931 (5118), Control: 4654 (1422), No CAR Control: 5381(3282))(Teff: MP: 1407 (288), Control: 521 (287), No CAR Control: 571 (220), p (MP vs Control and MP vs No CAR Control) < 0.001 for both), **(C)** CCR7 (Tcm: MP: 1152 (355), Control: 3784 (4601), No CAR Control: 578 (201), *p* (MP vs No CAR Control) = 0.003) (Teff: MP: 10919 (5421), Control: 8916 (2030), No CAR Control: 6468 (1484)) and **(D)** CD62L (Tcm: MP: 7268 (1437), Control: 13513 (10744), No CAR Control: 6655 (1635)) (Teff: MP: 6535 (1842), Control: 5430 (1122), No CAR Control: 2818 (994), *p* (MP vs No CAR Control) < 0.001, *p* (Control vs No CAR Control) = 0.005) in CD5+/CD45+/TCR-/transgene+ splenic lymphocytes are depicted on UMAP plots, representative histograms and bar graphs for Tcm and Teff. 2 donors, $N_{MP} = 10$, $N_{Control} = 6$, $N_{NoCARCtrl} = 6$. Error bars represent mean and standard deviation. Statistical significance was determined with a Brown-Forsythe and Welsch ANOVA test using Dunnett's T3 test for multiple comparisons; *p<0.05; ***p<0.001; ****p<0.0001.

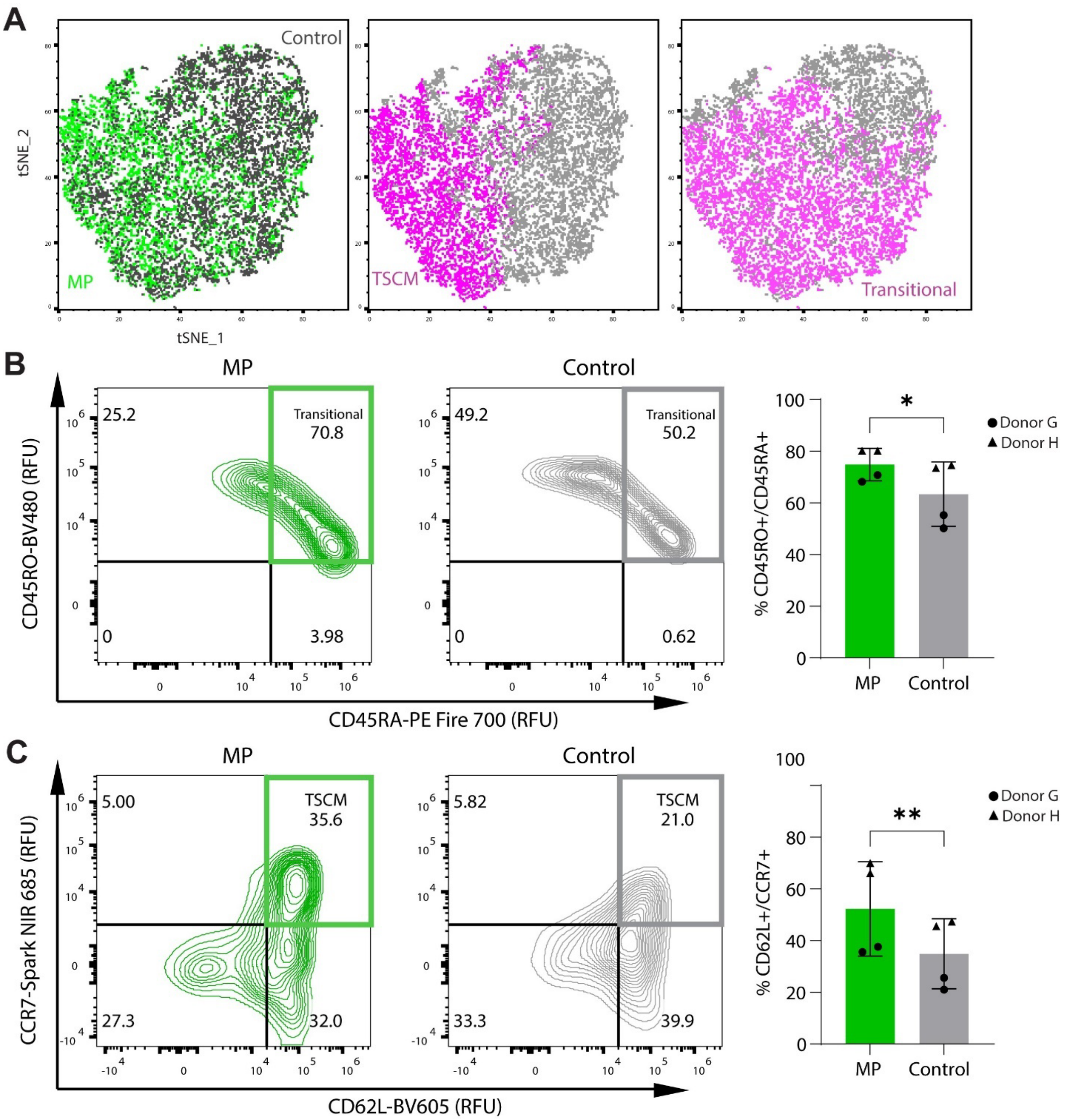

Figure S10. Differentiation of *TRAC*-CAR T cells in Serial Stimulation Assay. MP and Control *TRAC*-CAR T cells were serially stimulated with $GD2^+$ CHLA-20 neuroblastoma cells for 20 days, collected, and stained to immunophenotype human T lymphocytes via flow cytometry. **(A)** Marker expression t-SNE (t-distributed Stochastic Neighbor Embedding) plots of MP and Control *TRAC*-CAR T cells. These maps were generated via flow cytometry to track CD45RA, CD45RO, CD62L, CCR7, LAG3, and TIGIT expression. Dot plots separate cells by condition, T_{SCM}, or transitional T-cell status. Representative contour plot and bar graphs of double positive populations of **(B)** CD45RO vs CD45RA and **(C)** CD62L vs CCR7. 2 donors, $N_{MP} = N_{Control} = 4$. Error bars represent mean and standard deviation. Statistical significance was determined with paired t-tests; *p<0.05; **p<0.01.

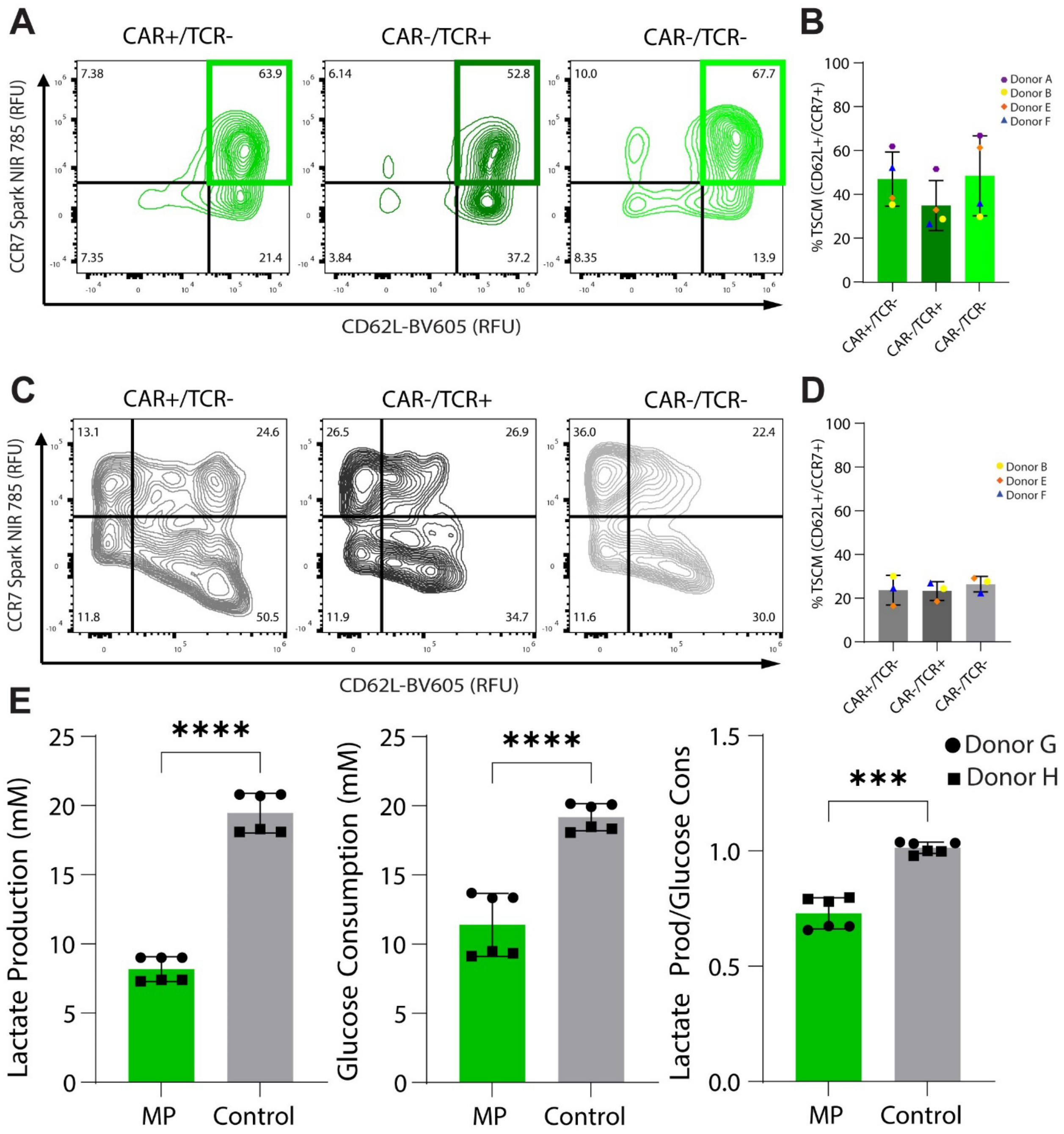

Figure S11. Expression of Stem Cell Memory Markers in Different T-cell Populations. Representative contour plots of CD62L vs CCR7 expression and bar graphs of double positive percentages in CAR$^+$/TCR$^-$, CAR$^-$/TCR$^+$, or CAR$^-$/TCR$^-$ populations of **(A,B)** MP (CAR$^+$/TCR$^-$: 47% (12), CAR$^-$/TCR$^+$: 35% (11), or CAR$^-$/TCR$^-$: 48% (18)) or **(C, D)** Control T-cells (CAR$^+$/TCR$^-$: 24% (7), CAR$^-$/TCR$^+$: 23% (4) , or CAR$^-$/TCR$^-$: 26% (4)). **(E)** Untransfected T-cells were grown for 10 days under MP or Control culture conditions where the lactate production (MP: 8.2 mM (0.9), Control: 19.5 mM (1.4), $p < 0.001$), glucose consumption (MP: 11.4 mM (2.3), Control: 19.2 mM (1.0), $p < 0.001$), and the lactate production over glucose consumption (MP: 0.73 (0.07), Control: 1.01 (0.03), $p < 0.001$) was measured on Day 10. Error bars represent mean and standard deviation. Statistical significance was determined with paired t-tests; ***p<0.001; ****p<0.0001.

Table S1. Guide RNA's and Primers Used in Study. The TRAC gRNAs and forward and reverse primers for amplifying the original GD2-CAR and No CAR Control linear constricts for nanoplasmid construction. (5' --> 3')

Oligo	Sequence
TRAC gRNA	CAGGGTTCTGGATATCTGT
TRAC PCR REV primer	TAAGGCCGAGACCACCAATCAG
TRAC PCR FWD Primer	TCGAGTAAACGGTAGTGCTGGG

Table S2. Nanoplasmid Sequences Used in Study. The full GD2-CAR and No CAR Control nanoplasmid DNA sequences are shown (5' --> 3') (in Excel addendum)

Table S3. Sanger Sequencing Primers Used in Study. The primers used for performing Sanger sequencing on the GD2-CAR or No CAR Control plasmid are shown. (5' --> 3') (in Excel addendum)

Table S4. Antibodies Used in Study for Flow Cytometry. The clone, manufacturer, fluorophore, catalog number, and volume needed per sample are listed for each antibody used for flow cytometry in this study. (in Excel addendum)
